# Intersegmental Eye-Head-Body Interactions during Complex Whole Body Movements

**DOI:** 10.1371/journal.pone.0095450

**Published:** 2014-04-24

**Authors:** Christoph von Laßberg, Karl A. Beykirch, Betty J. Mohler, Heinrich H. Bülthoff

**Affiliations:** 1 University of Leipzig, Institute of General Kinesiology and Athletics Training, Leipzig, Germany; 2 University Hospital Tübingen, Department of Sports Medicine, Tübingen, Germany; 3 Max Planck Institute for Biological Cybernetics, Department for Human Perception, Cognition and Action, Tübingen, Germany; 4 AMST Systemtechnik GmbH, Ranshofen, Austria; 5 Korea University, Department of Brain and Cognitive Engineering, Seoul, South Korea; University of Rome Foro Italico, Italy

## Abstract

Using state-of-the-art technology, interactions of eye, head and intersegmental body movements were analyzed for the first time during multiple twisting somersaults of high-level gymnasts. With this aim, we used a unique combination of a 16-channel infrared kinemetric system; a three-dimensional video kinemetric system; wireless electromyography; and a specialized wireless sport-video-oculography system, which was able to capture and calculate precise oculomotor data under conditions of rapid multiaxial acceleration. All data were synchronized and integrated in a multimodal software tool for three-dimensional analysis. During specific phases of the recorded movements, a previously unknown eye-head-body interaction was observed. The phenomenon was marked by a prolonged and complete suppression of gaze-stabilizing eye movements, in favor of a tight coupling with the head, spine and joint movements of the gymnasts. Potential reasons for these observations are discussed with regard to earlier findings and integrated within a functional model.

## Introduction

The human visual system is critical to spatial orientation and controlled movement, which require the brain to continuously and precisely adjust the eyes. Eye movements represent the computational output resulting from multiple sensory inputs, their context specific weighting and additional influences from motor learning and cognition. Therefore, the study of eye movements is a highly relevant approach to better understand general principles of interactions between perceptual and motor control mechanisms in humans. In this paper, we describe for the first time measuring sequences which are able to integrate synchronous capturing of intersegmentalspinalmotor data, neuromuscular activation, and eye movements during actively performed multiaxial whole body movements.

The most studied eye movements in awake individuals are those that serve to stabilize objects in the environment on the retina to avoid blurred vision. Although several systems are involved, for the purpose of this paper, we call all of these “environment-referenced eye movements” (EREMs). We further distinguish EREMs as being compensatory, to compensate for head movements in space and keep the eyes stable in space, or non-compensatory, if they do not stabilize the eyes in space. Compensatory eye movements use sensory feedback about the head's motion to reflexively move the eyes in the opposite direction. When the sensory system is the inertial-motion sensor in the inner ear, the vestibular system, the result is termed the vestibulo-ocular reflex (VOR). When vision itself uses large-field optical motion to stabilize the eyes, the optokinetic reflex (OKR), keeps the large-field image stable on the retina. Head motion that would require too large an eye movement results in a fast, resetting motion, interspersed with the slow compensatory motion. This sawtooth-formed motion is called *nystagmus*, whether it is produced by VOR or OKR. Both vestibularly driven (VOR) and visually driven (OKR) eye movements can result in nystagmus. Nystagmus due to OKR is called optokinetic nystagmus (OKN).

Whether or not the head is in motion, the volitive tracking of an object that moves relative to the head is accomplished with the smooth pursuit eye movement (SPEM) system. SPEM keeps the object of interest on the central, high-resolution area of the retina, the fovea. Because the purpose is smoothly pursuing or tracking the object, not compensation for head movements, it is typically non-compensatory. However, it is possible during slower head movements to intentionaly watch a stationary target, activating the SPEM in a *compensatory* fashion. Another non-compensatory movement occurs when the object of interest is not yet on the fovea and the eyes need to be redirected to the new focus of attention. Such a fast motion of the eyes causes blurring of the image, so they move as quickly and accurately as possible to minimise loss of vision. This intentional redirection of the eyes is known as a saccade.

Much less studied and much more difficult to interpret are eye movements most likely induced by proprioceptive stimuli, e.g., the cervico-ocular reflex (COR), the trunk-ocular reflex (TOR), the arthrokinetic response (AKN [similar to OKN, the abbreviation AKN results from “arthrokinetic nystagmus”]), and the smooth pursuit of non-visual objects (e.g., following one's own arm movements in the dark or even by pure imagination) [1]–[12].

The main purpose of the COR is often described as supporting VOR and OKR functionally by a compensatory response to head movements [4]. However, authors who conducted experiments of passive body movements made in the dark while the head was fixed (to exclude VOR) have described the COR (and also the TOR) as weak reflexes that normally move the eyes in the *same* direction as the head or torso in healthy persons [5]–[8]. Therefore, some authors describe these as “anti-compensatory” eye movements, meaning directed exactly opposite to compensatory motion. Here we distinguish between eye movements that are compensatory and those that are not, and use the term “non-compensatory” for all that are not. This primarily non-compensatory direction of COR may also reverse direction to provide compensatory responses (e.g., if the vestibular system is compromised because of injury or disease) [9], [10]. This underlines the general priority of the compensatory functions of gaze stabilization during motion; however, the role of the non-compensatory components of these reflexes is unclear. Some authors [7], [11] have described a further kind of reflexive oculomotor response in darkness, induced by passive leg rotation in a chair while the rest of the body remained stationary. However, in contrast to the non-compensatory COR and TOR direction, this response was described as compensatory. In this way, the leg-induced response seems to be similar to the AKN of the arm [12]. These most likely proprioceptively-mediated oculomotor responses seem quite puzzling and inconsistent, and their purpose is not entirely clear.

There have been multiple studies concerned with eye-movement characteristics during passive whole body movements and during active skills, with regard to their roles concerning spatial representation and motor control [13]–[17]. Other studies have referred to specific adaptation of eye movement characteristics in elite athletes to efficiently master context specific skills [18], [19].

Previous work regarding reflexive eye movements in people experienced with *rotational loads* have investigated differences between novices and experts like fighter pilots [20]–[22], figure skaters [23], [24], or gymnasts [25], [26]. Such studies have mostly referred to questions of VOR adaptation and its effects with regard to human spatial orientation. To facilitate comparison, such investigations usually involve standardized, passively induced rotational motions. Whereas the phenomenon of habituation of the VOR in populations experienced with rotational loads has been well documented [20], [22], [25]–[28], their relationships with the accuracy of VOR characteristics (VOR gain [quotient of eye velocity/stimulus velocity] and temporal phase shift to the stimulus) seem to be inconsistent and not completely clear. For example, Lee [21] and Schwarz & Henn [22] described higher VOR gain values in rotationally habituated pilots. However, Ahn [20] could not find such differences between pilots and controls. In contrast, Tanguy et al. [24] observed significant gain reductions, combined with phase advance in figure skaters. According to this, Clement et al. [28] also described a VOR-phase advance after repeated vestibular training (velocity steps) of normal healthy persons, but in contrast there were no changes in VOR-gains. Some authors assume that these diverging kinds of rotational VOR adaptations are caused by highly context specific cues [23], even though the detailed mechanisms responsible for such contrary findings are not completely identified.

These studies provide evidence for experience-related changes in the processing of reflexive eye movement characteristics. However, most of these studies were conducted under passively driven laboratory conditions. Thus, more context-specific cues that could relate to real-life scenarios during actively performed whole-body rotations are not adequately represented in these approaches.

With respect to questions of visual perception, however, some studies were conducted under actively performed conditions with gymnasts, using new lightweight eye tracking systems during somersaults. These studies addressed the function of visual control within the framework of visual areas of interest that are necessary to optimize performance, in particular a safe landing, rather than investigating the underlying mechanisms, reflexive or otherwise [29]–[33]. Consequently, it could be assumed that individual improvement in OKR and/or SPEM velocities could be beneficial for gymnasts. In this respect, our own studies confirmed this hypothesis by showing a significant enhancement of SPEM performance in gymnasts compared with non-athletes [34].

In summary, there have been multiple studies of passive whole body movements and a few of active performance of airborne, whole-body rotations. However, until now there have been no descriptions of specific eye movement characteristics under highly complex conditions, like the performance of multiple twisting somersaults. Furthermore, no data are available that can elucidate the relationships between eye, head and body movements during such complex skills, nor their relationship to intersegmental neuromuscular activation during such conditions. The present study was intended as a first step in this direction.

In preparation for this study, different test series and pilot studies were conducted to get a first assessment to this approach and to optimize the experimental setup over years [25], [26], [34], [35]. Some unexpected observations should be mentioned:

1. During actively performed monoaxial and multiaxial somersaults, complete nystagmus suppression was observed, whereas the same participants did show an intense nystagmus response during passively driven rotations with similar velocities [26], [35].
2. No other oculomotor functions (e.g., SPEM) were observed during these nystagmus suppression phases [35].
3. During these phases, however, specific relationships of non-compensatory coactivations between eye movements and body movements were observed [35].

These novel observations led to the hypothesis that during actively performed whole-body rotations, a neural mechanism that suppresses the EREM and is tightly associated with intersegmental body movements becomes dominant [35]. Thus, in addition to giving a general description of oculomotor behavior during actively performed twisting somersaults, a specific aim of this study was to verify whether this hypothesis can be statistically supported with data from a group of high-level gymnasts.

## Materials and Methods

### Ethics statement

All measurements were conducted according to the principles expressed in the Declaration of Helsinki and were undertaken with the understanding and written informed consent of each subject or their parents, in case of juvenile participants. This includes consent for publication of their photograph, as outlined in the PLOS consent form for publication in a PLOS journal. The study was supported by the German Institute for Sport Science and was approved by a decision of the German Parliament. It was not required to obtain approval by an additional institutional review board for this study. This was confirmed by a written waiver of the German Institute for Sports Science. No research was conducted outside of our country of residence.

### Participants

Seven male gymnasts aged 16–28 years were recruited from the National Training Center for Artistic Gymnastics in Stuttgart: 2 members of the German National Team, 2 aspirants to the national team and 3 state junior gymnasts.

### General description and procedure of the measurements

After preparation with EMG electrodes and markers for kinematic capture, “maximum voluntary contraction” (MVC) measurements of all the muscles prepared with the EMG electrodes were recorded. Then a “range of motion” measurement was recorded during motion of all of the body joints that were measured, for adjusting the kinematic data to the individual proportions of the subjects. All joints and angles that were analyzed in this study are listed in Table 1. Next, the goggle of the oculography system was fixed on the head of the participants. After calibration of the oculography system the athletes had to perform stretched somersaults from a trampoline, and then land on a landing mat. A “streched somersault” is the official term in gymnastics meaning the body is held as straight as possible, as opposed to “tucked somersault”, for example. Each participant had to begin with simple streched somersaults without superposed longitudinal axis rotations (LAR), and then enhance the level of difficulty, trial by trial, performing somersaults with increasing numbers of LARs, depending on his individual technical ability. Each element a participant could perform should be done twice. All trials were included in the statistical analysis, with exception of trials with failures caused by the gymnasts (such as a fall) or failures caused by technical reasons such as lost markers, foggy goggles or any disturbances of signal transmission.Each measurement included a calibration procedure of the eye tracking system (see later in the text) and a set of three somersault trials. The participants took a break between the measurements for approximately 2–3 minutes. During this time, the experimenter named and stored all measurement files and visually determined that all of the equipment (markers, electrodes and wires) were still well fixed. This was important to prevent any signal failures in subsequent trials.

**Table 1:** Definition of kinematic variables.

Variables	Definition
**Eye movements**	
Horizontal	Angle of horizontal eye movement components in the head (right: +; left −)
Vertical	Angle of vertical eye movement components in the head (up: +; down: −)
**Head movements**	
Pitch axis	Angle between head and trunk segment (horizontal axis; up: +; down: −)
Yaw axis	Angle between head and trunk segment (vertical axis; right: +; left −)
Roll axis	Angle between head and trunk segment (sagittal axis; right: +; left −)
**Lumbar spine movements**	
Sagittal flection	Angle between trunk segment and pelvis segment in the sagittal plane (Extension: +; Flexion: −)
Lateral flection	Angle between trunk segment and pelvis segment in the frontal plane (to the right: +; to the left: −)
**Leg-trunk movements**	
Sagittal flection	Angle between the upper leg segment and the trunk segment. This includes the lumbar spine angle plus the hip angle (pelvis segment to upper leg segment) in the sagittal plane (Extension: +; Flexion: −)
Lateral flection	Angle between the upper leg segment and the trunk segment. This includes the lumbar spine angle plus the hip angle (pelvis segment to upper leg segment) in the frontal plane (to the right: +; to the left: −)
**Knee movements**	
Knee Flection	Angle between the upper leg and the lower leg in the sagittal plane (Flexion: +; Extension: −).

Altogether, 76 trials were analyzed (Table 2): somersaults *without* superposed LARs backward and forward, somersaults with backward takeoff with 0.5 LAR, 1.0 LAR, 1.5 LARs up to 2.0 LARs, and somersaults with forward takeoff with 0.5 LAR, 1.0 LAR, 1.5 LARs, 2.0 LARs up to 2.5 LARs. The direction of the LARs was individually different: 4 participants were turning to the left, 3 were turning to the right.

**Table 2:** Analyzed trials.

	Participants							
Elements	P1 (ST)	P2 (LB)	P3 (OT)	P4 (AU)	P5 (KM)	P6 (KE)	P7 (TA)	Trials per element
Sfwd 0	1	2	-	1	2	1	1	n = 8
Sfwd 0.5	1	-	1	1	2	2	-	n = 7
Sfwd 1.0	1	2	-	2	-	2	1	n = 8
Sfwd 1.5	2 (2)	2 (1)	2 (2)	2 (2)	2 (1)	1 (1)	2 (1)	n = 13 (10)
Sfwd 2.0	1 (-)	-	-	-	-	-	2 (-)	n = 3 (-)
Sfwd 2.5	-	2 (2)	1 (1)	-	-	-	-	n = 3 (3)
Sbwd 0	1	2	2	1	2	1	2	n = 11
Sbwd 0.5	1	-	-	1	-	1	-	n = 3
Sbwd 1.0	1 (1)	-	2 (1)	2 (1)	2 (2)	1 (1)	-	n = 8 (6)
Sbwd 1.5	1 (1)	1 (1)	-	-	-	2 (2)	-	n = 4 (4)
Sbwd 2.0	2 (2)	1 (1)	2 (1)	1 (1)	2 (2)	-	-	n = 8 (7)
**Trials per subject**	n = 12 (6)	n = 12 (5)	n = 10 (5)	n = 11 (4)	n = 12 (5)	n = 11 (4)	n = 8 (1)	total n = 76* (30)

*76 trials were included for the descriptive analyses. Only the numbers in parentheses were included for the bivariate statistics of correlations between eye, head and body movements.

The measurements of kinematic, electromyographic, and videonystagmographic data were taken with a synchronous capture, triggered via an optical or analog signal for subsequent temporal synchronization. After post-processing, the data sets were integrated, visualized, and prepared for further statistical analysis by a custom-made software tool “Vismo” [36], developed for the special requirements of analysis of the interactions between eye-, head- and body movements and neuromuscular activation during highly dynamic motion sequences in sports. Based on the 3D data of the head position in space and eye position in the head, the direction of the participants' gaze line was calculated.

### Measurement components and data processing

#### Kinematic data

All somersaults and measurements were performed in the large motion capture laboratory at the Max-Planck-Institute for Biological Cybernetics in Tübingen. This laboratory has a 16-channel infrared kinemetric system (Vicon, USA) in a 12×12×8 (height) meter space. The system works with 16 active infrared cameras (Vicon, MX13) detecting reflective markers on specific body parts of the participants. The head was prepared with 5 markers to assure the virtual reconstruction of the exact head position at all times. The body was prepared with an additional 16 markers over standardized locations at the ankles, knees, hips, pelvis, shoulders (each at the acromion and over the upper teres major for also being able to precisely reconstruct the shoulder in overhead position of the arms), elbows and wrists. To generate a complete lattice model of the body in Vismo without any gaps, the captured scatter plots of the markers had to first be post-processed carefully by a manual labeling procedure (Software: Vicon IQ 2.5).

The specific challenge of capturing very fast, dynamic and complex movements meant that it could not be guaranteed that during specific movement phases all the reflective markers were constantly visible by enough infrared cameras for precise spatial reconstruction. Therefore, an independent 3D video capturing system (3D-Mess - a custom made software, developed for sport specific analyses by the Institute of Applied Training Sciences in Leipzig) was used to additionally capture the movements to ensure that all the movements could be reconstructed completely and precisely. 3D-Mess relied on data from two synchronized video cameras (master camera: Canon XM1; slave camera: Panasonic WV-F15; both 50 frames/sec), positioned in a right angle, from the side and the front of the movements main direction. To enable the complete reconstruction of the whole movement the markers of any missing body parts could then additionally be labeled manually, frame by frame, and then manually inserted into the infrared data files. To enable this data compatibility, both the infrared data and the 3D-video data, were transformed to a mutual data file format (RKA-format) before the integration and further use of the file in Vismo. However, in only very few cases (some frames in 3 or 4 trials) this hybrid procedure was necessary, because the movements could be mostly reconstructed by the infrared data alone, if at all. The kinematic variables used for further analysis were defined as follows (Table 1).

#### Electromoygraphic data

Wireless surface electromyography (EMG) was captured at 1500 Hz (Telemyo 2400T, Noraxon, USA). The trigger signal was recorded on a separate channel of the EMG recording. Furthermore, an elastic bodysuit was worn to affix the transmitters, electrodes, and cables tightly to the body, in order to not disturb the athletes' movements. The EMG preparation was in accordance with the “Standards of reporting EMG data” by the International Society of Electromyography and Kinesiology [37].

After shaving and fine grinding the skin area with mild sandpaper, a pair of gel covered AG/AL disc electrodes (Ambu blue sensor, Denmark; radius: 1 cm) was placed with an inter-electrode distance of 2 cm in alignment with the fiber direction. A reference electrode was attached to the sternum. All electrodes were fixed on the skin with tape to avoid movement artifacts. Electrode skin impedance was accepted at a level of <5 kOhm. The following muscles were captured: *pectoralis major* (Pect), *rectus abdominis* (RAbd), and *rectus femoris* (RFem) as essential parts of the anterior muscle chain, and *deltoideus* (Delt.), *erector spinae* (ESpin), and *biceps femoris* (BFem) as antagonists (posterior muscle chain). The following standards of EMG detection were given by the manufacturer: Input impedance: >100 MOhm; CMRR: >100 dB, SNR: Baseline noise <1 µV RMS. The raw EMG signals were bandpass filtered (10 to 500 Hz) and sampled with 1500 Hz, AD-converted (12 bit) and stored for further processing on a PC-System. Using Noraxon Software (Myoresearch, Version 1.06.60; Noraxon, USA), the raw data were full-wave rectified and smoothed over a constant time window of 25 ms.

#### Oculomotor data

To capture the oculomotor data, a custom-made sports video-nystagmographic goggle system (VNG) was developed in close cooperation with Interacoustics (Denmark), Chronos Vision (Berlin, Germany), and Plexiglas Kienzle (Stuttgart, Germany). This lightweight goggle (200 g) allowed for binocular vision of the participant with a wide field of view (horizontal: 138°; vertical: 66°). A small infrared eye camera (50 Hz) with wireless data transfer was integrated inside the case of the goggles to view the left eye by an infrared mirror that was visually transparent. The area in front of the eyes was covered by a special infrared-transparent shield of plexiglass to protect the eyes in case of a fall. The goggles were constructed to fit as tightly as possible on the subject's head. Since it was not possible to avoid goggle movement relative to the head during high accelerations, the detection software included an additional capture of the position of the eye inner canthus (inner edge of the eye lid angle) as a reference for goggle movements. Based on the canthus position, the detected pupil position could be corrected with a semiautomatic algorithm in the post-processing procedure. Within this procedure, artifacts of automatic pupil detection during high accelerations could also be manually corrected. Both the individual nonlinearity of the eye movements (depending on the size of the bulb and the distance to the infrared mirror) and the individual kinetics of the canthus at different eye positions were detected during the calibration procedure for later inclusion in the algorithm for the gaze line calculation in Vismo. The calibration procedure was conducted with 4 infrared reflecting markers in a formation of a 2×1 meter sized rectangle with a fifth marker placed in the center of the rectangle. This rectangle was fixed on a vertically adjustable canvas (precisely located 5 meters from the participant), which was located at the edge of the workspace. The middle marker was precisely adjusted to the height of the participant's eyes during a clearly defined upright standing position. At the beginning of each measurement (each including a set of 3 somersaults), the common trigger signal for later synchronization of all the measurement systems was manually activated and the participants had to first fixate each of the four corner markers (one after the other) without moving the head, and then visually focus on the central marker during first horizontal and then vertical head movements. After the calibration procedure, the participants began to perform their somersaults. Following each set of three somersault trials, the participant again fixated the central marker and an additional trigger signal was activated.

#### Integration and treatment of the data components in Vismo

The trigger signal for synchronization of the different data components produced a frame-based signal in the data of all capturing systems (Vicon, EMG, VNG). After integration of all data components into the software Vismo, the data were synchronized and calibrated for creating a virtual model of the athlete.

The virtual room of the workspace was adapted exactly to the real dimensions of the lab, including the gymnastic apparatuses and the canvas with the markers used for calibration.

The software generates a 3D, 15-segment lattice model of the subject, based on the kinematic data. Gaze line and gaze spots were calculated and adjusted exactly to the rectangle of the canvas for calibration, depicted in the virtual space (a “gaze spot” in the sense of this study is defined as the location, were the gaze line meets the environment). The lattice model was smoothed over 11 frames (20 ms each) and the eye data was smoothed over 3 frames by a Gaussian function. This specific combination of smoothing procedures has been identified to generate the best results for most realistic virtual depiction and further calculations (unpublished material of a project, supported by the German Institute for Sports Science, 2010 [AZ: IIA1-070605/08-09]).

**Figure 1:**
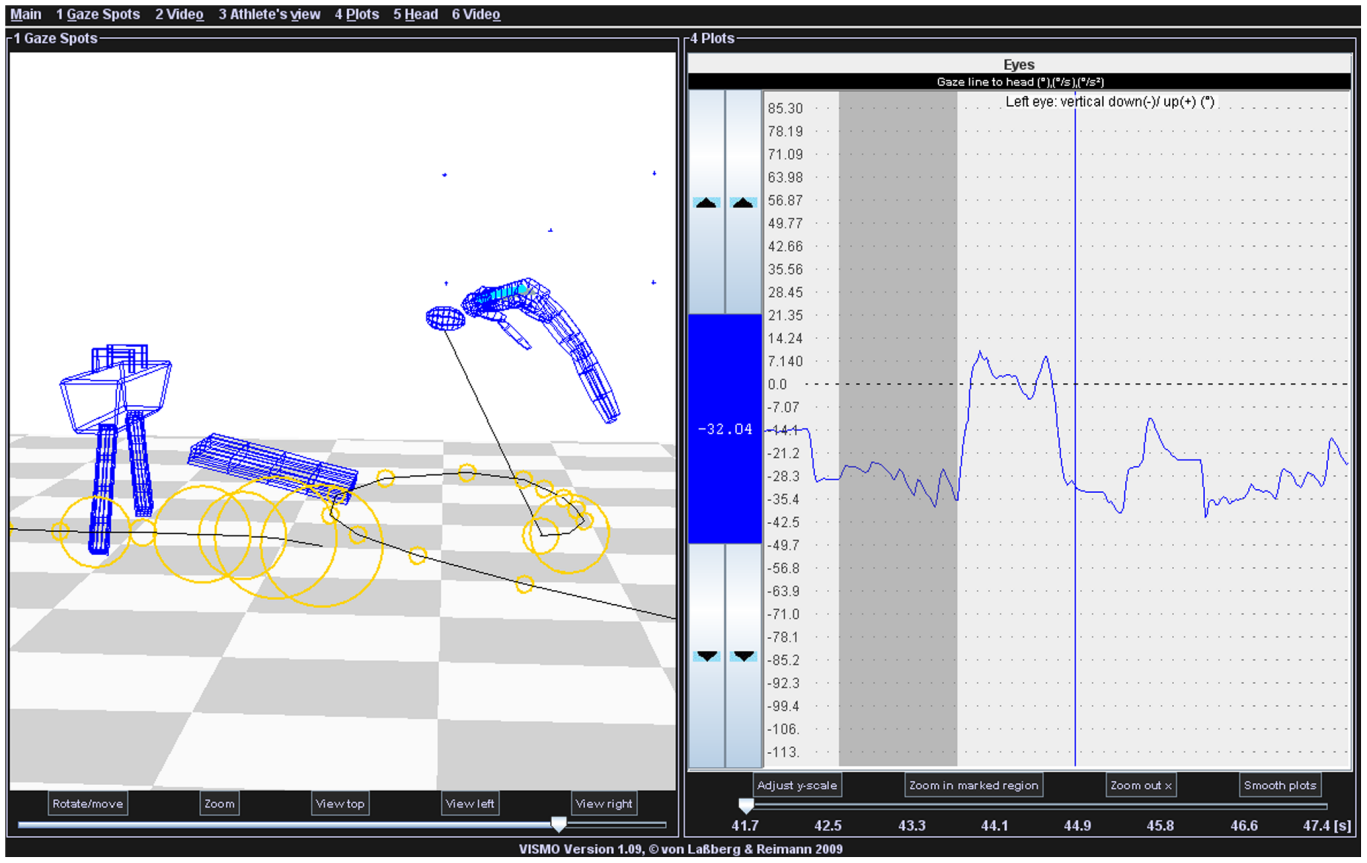
Eye movements during twisting somersaults. Screenshot of Vismo software showing analysis of an element just before landing during a double twisting somersault. The vertical line in the plot on the right represents the moment of the current frame in the virtual room (on the left). Left: Lattice model of the virtual room and gaze spot function for visualization of eye movements, marked by different sized circles. The gaze spot function is described in the Methods. To summarize, the size of the circle shows the duration of looking at that spot (the center). Small circles may show fast components of nystagmus or saccades, but in this case, multiple small circles indicate a period without EREM (most of them are located outside of the field of this present virtual view), that is a “suppression phase”. In the background of the work space, the rectangle with the 5 markers for the oculomotor calibration procedure can be identified. Right: Concurrent view of the vertical components of the eye movement plots (the synchroneous plot of horizontal eye movements is not depicted in this view). The highlighted section at the beginning shows the nystagmus activity, before the contact with the trampoline (see also: Video S1). The end of the highlighted section marks the moment of take-off and the beginning of the “suppression phase”, which corresponds on the left to the series of small circles joined by a curve.

A special “gaze spot visualization function” represents the resulting coordinates of the central visual target in the environment, calculated by the head-related eye data (corrected by the canthus data; see above) and the 3D head position in the space. The gaze areas are represented by small circles around the viewed point of the surrounding surfaces. If the calculated gaze line remains at the same point for several frames, the diameter of the circles in the virtual environment grows, frame by frame, as a linear function of the visual fixation time (Fig. 1 and Video S1). During fast eye movements (e.g., saccades) or gaze movements, which were not related to any environmental structure, only small circles are depicted, representing the current gaze spot of the corresponding frame. A fixed value, such as 3°, is often used to define the maximum range of deviation during visual fixation sections, meeting the requirements for visual information processing [2], [18]. For the determination of the maximum range of deviation (to be interpreted as “the same point” during visual fixation) we generally based the setting for the depiction of growing circles on the above mentioned 3°. However, to assure a correct adjustment that also considers differences of the eye data quality between different measurements we could manually adjust the settings within the calibration sequence by ensuring that growing circles occurred while viewing each point of the calibration rectangle, especially the central point during the head movements.

This “gaze spot visualization function” helps to distinguish EREMs from phases where EREMs are not apparent.

#### Data analysis

**Figure 2:**
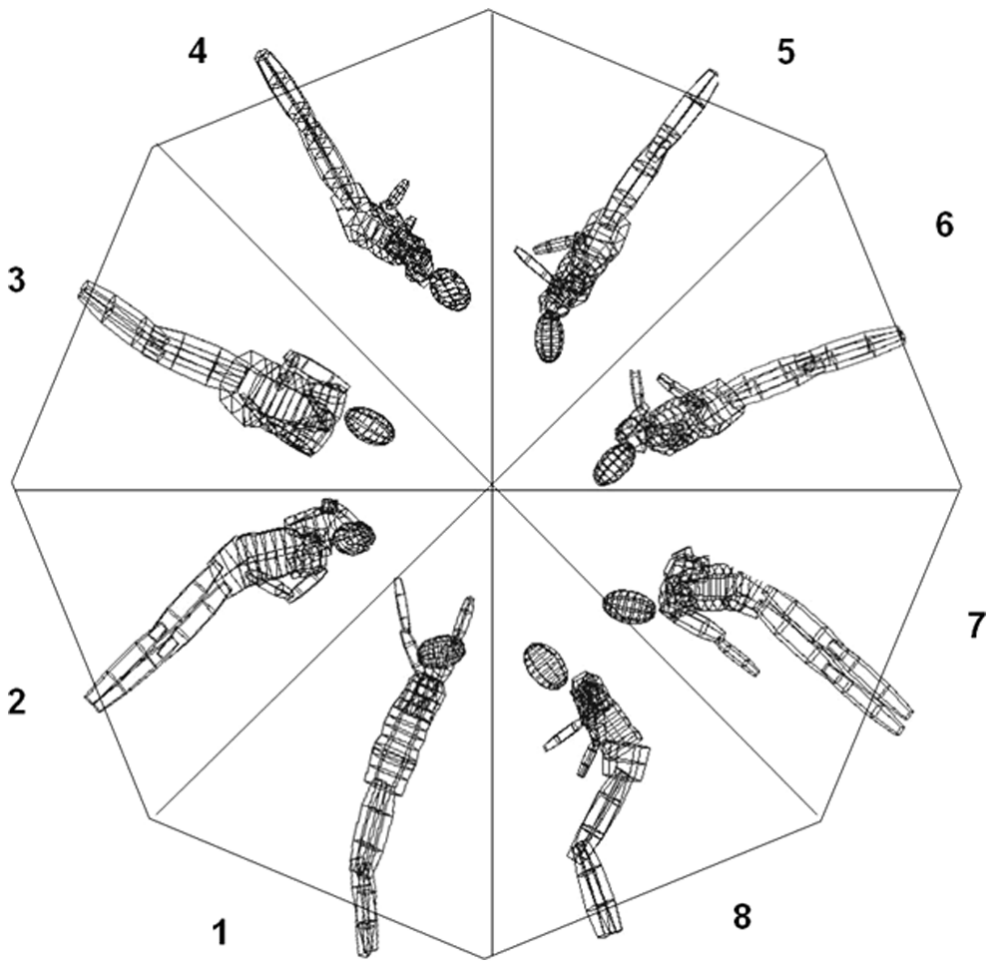
Sectors for descriptive data analysis. Demonstration of the 8 sectors used for desciptive analysis (see text for details).

For the *descriptive* data analysis of the eye movements for each element, the somersault was divided into eight 45° angular sectors of the 360° rotation (Fig. 2). The transition between sectors was determined using the straight line between the shoulders and the ankles. The definition of angular sectors was chosen to allow objective comparison between subjects, since the 360° rotation was the only aspect that was identical for each trial. Other possible methods such as defining functional sectors (e.g. take off phase, twisting phase etc.) or breaking the duration into equal time segments, although often strongly related to the angular sectors, did show inter-subject, inter-trial and inter-element variability that we sought to avoid.

Three steps were taken to analyze the interaction of eye and body movements. First, each of the 8 sectors (see Fig. 2) was analyzed by visual inspection of the horizontal and vertical eye movement plots and via the “gaze spot visualization function” of the software (see Fig. 1) for counting the number of frames showing eye blinks (marked by a lid closure with interruption of the gaze line calculation), the number of frames showing fast phases of EREMs (nystagmus beats and saccades) and the number of frames showing slow phases of EREMs. Throughout this analysis, all EREM phases during the movement were determined. For further details, how these phases are defined exactly and how they are detected using the “gaze spot visualization function” of the software, see Table 3.

**Table 3:** Definitions of types and components of EREM**.

Types	Compensatory components		Non-compensatory components	
	Definition	Marked in the software with:	Definition	Marked in the software with:
1. Smooth Pursuit Eye Movement (SPEM)	Intentionally watching an object that moves relative to the head. It may be compensatory if the relative motion is due to slow head movement with a stationary target.	Growing circles	Intentionally watching an object that moves relative to the head. It is non-compensatory whenever the object itself moves.	(does not occur - there are no moving objects in the room during the experiment)
2. Gaze stabilizing reflexes (e.g. VOR, OKR; see text), which may result in nystagmus (slow phases interrupted by fast resetting phases)	The slow phases of involuntary (reflexive) eye movements that keep a stationary surround stable on the retina during head motion. These reflexes may be suppressed by SPEM.	Growing circles	The reflexive fast, short, linear resetting movements, accompanying and counterdirected to the slow phases*. Vision is suppressed during fast phase, as they would merely be a blur. These reflexes may be suppressed by SPEM.	A fast component* results in a new small circle. If the fast component is less than 20 ms (one frame), the small circle grows because a new slow component has started. If the fast component lasts longer than 20 ms, multiple small circles show the single-frame gaze position during that frame.
3. Saccades	(Saccades are non-compensatory)	----	Saccades are fast redirection of the eyes to a new target. They are similar to fast nystagmus phases*, but often last longer. They can be reflexive or intentional.	The same as the fast components of nystagmus.*

*Fast phases (nystagmus beats/saccades) were additionally confirmed and counted by a detailed visual inspection of the horizontal and vertical eye movement components for each of the 8 sectors.

**Definition “EREM”: We have defined EREM as: all phases with growing circles, which may be interrupted by saccades/nystagmus beats (sets of growing circles, perhaps interrupted by small circles if they were longer than 20 ms, i.e. one frame). **Definition “Suppression phase”**: We have defined “suppression phases” as: all phases that don't have EREMs and that, additionally, last a minimum of 2 sectors. This minimum duration guarantees a clear differentiation between “suppression phase” and very large saccades. Because the flight durations lasted between 800 ms and 1000 ms, the duration of 1 sector is about 100–125 ms and 2 sectors last about 200–250 ms. Even very large saccades do not last so long (Leigh & Zee, 2006; pp: 111–112) [39].

Second, if sections that lacked all of these patterns were found, showing only small circles in the “gaze spot visualization function”, lasting at least two sectors, they were defined as “EREM suppression phases” (Table 3). These sections were marked and separated in an extra file (synchronized with the kinematic and neuromuscular data), and finally exported for further statistical analysis.

Finally, the relationship between eye-, head- and body movements was examined to determine if there is evidence that a relationship between eye movements and head and body movements during these “suppression phases” really exists as predicted by our hypotheses. A correlation analysis of the eye data with the head, and body data during the exported “suppression phases” of each somersault was calculated (Pearson correlation coefficient). Additionally, the relationship of these kinematic data with EMG data was analyzed by the same procedure. For further evaluation of whether correlations found during the somersaults are simply caused by chance or if the correlations support real evidence for a systematic phenomenon, additional bivariate t-test analyses were conducted. These t-tests should reveal if the incidence of weighted positive and weighted negative correlations during the “suppression phases” across all trials of all participants are widely balanced (no evidence for systematic effects) or if positive or negative correlations are significantly prevalent (evidence for a systematic phenomenon). The software package “R” (R Foundation for Statistical Computing, Version 2.15.2; Vienna, Austria) was used to conduct the statistical analyses. The significance level was set at α = 0.05.

## Results

### Descriptive data analysis

**Figure 3:**
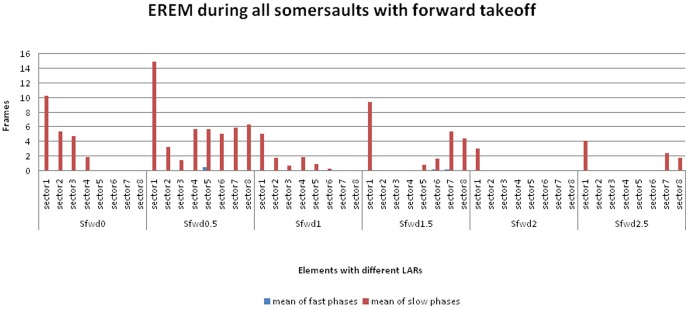
EREM during somersaults with forward takeoff. Histogram showing mean number of frames with fast and slow components of EREM during somersaults with forward takeoff. The mean number of frames showing fast and slow components for each element of forward somersault, varying with the number of longitudinal-axis-rotations (LARs) for 0 to 2.5 (Sfwd 0 to Sfwd 2.5). Within each element, the frames are shown by sector (see Fig. 2). While the take off is forward in *all* the trials, the body position during the landing differs according to the number of LARs. Landings forward: Sfwd 0; Sfwd 1. Landings backward: Sfwd 0.5; Sfwd 1.5; Sfwd 2.5. The number of trials and mean flight duration times (leaving the trampoline until first touch of the floor [M ± SD in ms]): Sfwd 0 (n = 8): 770±42.7; Sfwd 0.5 (n = 8): 858±104.44; Sfwd 1 (n = 8): 773±79.96; Sfwd 1.5 (n = 13): 903±103.89; Sfwd 2 (n = 3): 820±0; Sfwd 2.5 (n = 3): 980±52.92.

**Figure 4:**
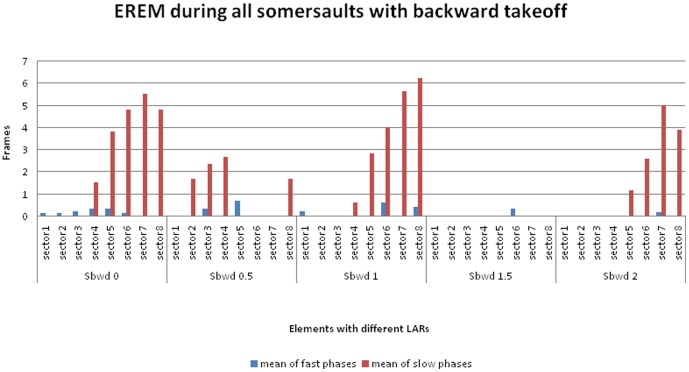
EREM during somersaults with backward takeoff. Histogram showing mean number of frames with fast and slow components of EREM during somersaults with backward takeoff. Similar to Figure 3, but with *backward* takeoff. While the takeoff is backward in *all* the trials, the body position during the landing differs according to the number of LARs. Landings backward: Sbwd 0; Sbwd 1. Landings forward: Sbwd 0.5; Sbwd 1.5. Number of trials and mean flight duration times (leaving the trampoline until first touch of the floor [M ± SD in ms]): Sbwd 0 (n = 10): 827±65.92; Sbwd 0.5 (n = 3): 733±30.55; Sbwd 1 (n = 8): 818±75.17; Sbwd 1.5 (n = 4): 750±66.33; Sbwd 2 (n = 8): 860±30.24.

**Figure 5:**
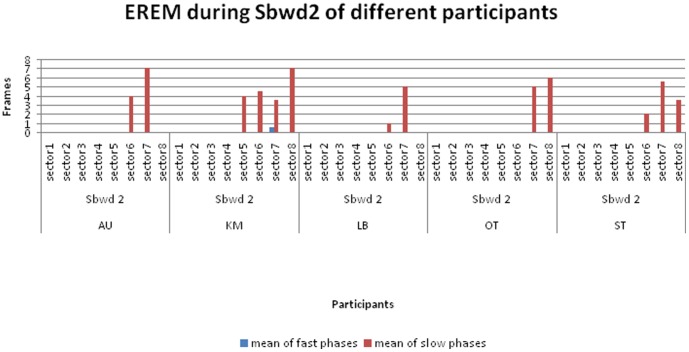
Example of inter-individual difference for EREM patterns during backward somersaults with 2 LARs. Similar to Figures 3 and 4, but showing sectors with EREMs of all backward double twisting somersaults (Sbwd 2; with a backward takeoff and landing). Number of trials and mean flight duration times (leaving the trampoline until first touch of the floor [M ± SD in ms]): AU (n = 2): 830±14.14; KM (n = 2): 890±14.14; LB (n = 1): 840; OT (n = 1): 900; ST (n = 2): 850±14.14.

EREMs were identified during nearly all somersaults (see Fig. 3–5, and Video S1).

For trials where suppression phases occurred, the duration of the suppression was strongly associated with the number of LARs, and the sectors in which they were found depended on the body direction during takeoffs and landings (see Figs. 3 and 4). These figures show that extended phases of EREMs were found in all participants during forward take offs and during preparation for backward landings (Fig. 3: Sfwd 0.5; Sfwd 1.5; Sfwd 2.5). In contrast, only a few EREMs were detected during backward takeoffs and forward landings (Fig. 4: Sbwd 0.5; Sbwd 1.5). Additionally, the more LARs that were performed, the fewer EREMs were detected in general (Fig. 3; Fig. 4). A comparison of the Figures 3 and 4 with Figure 5 demonstrates that the movement phases which are marked by EREM were more closely associated with the kind of the elements than with individual peculiarities of the athletes.

As defined in the Methods, all phases that were not marked by any EREM functions for a duration of at least two sectors were defined as EREM suppression phases. With the exception of some forward somersaults with 0.5 LAR (Sfwd 0.5) and some backward somersault without LAR (Sbwd 0), suppression phases occurred in every trial of all the elements with a minimum of 1 LAR (Fig. 3–5).

Eye blinks were observed only before takeoffs and after landings. Aberrant phenomena like closed eyes or deviation of the pupil under the upper eyelid, which was sometimes observed in lower-level gymnasts [35], were not be observed in our sample of high-level gymnasts.

### Interactions between eye movements and body movements during EREM suppression phases

**Figure 6:**
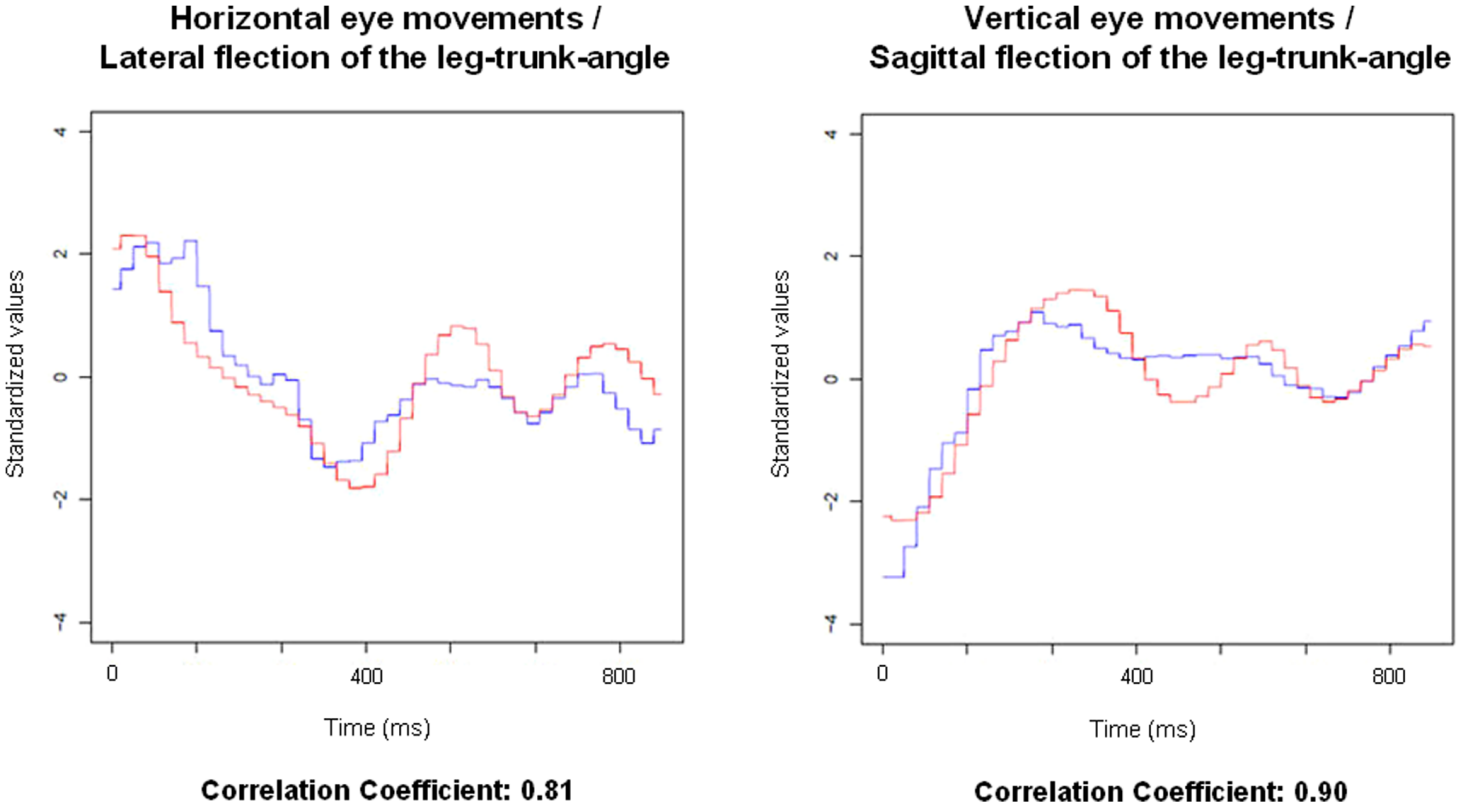
Angle-time characteristics. Examples of angle vs. time characteristics of eye and corresponding planes of body movements during the suppression phase of a double-twisting backward somersault and the Pearson correlation coefficients. The definition of the variables is summarized in Table 1.

We tested the hypothesis that during suppression phases, eye movements are associated with body movements (an example of such correlations is given in Figure 6). For this purpose we conducted a weighted, bivariate *t*-test analysis across all the suppression phases of all participants to determine if there was a significant difference in frequency of positive and negative correlations.

To ensure exclusion of simple monaxial eye-head saccades, we excluded those somersaults without an LAR and those with only short suppression phases, and selected only elements that consisted of multiaxial movements with a minimum of 1 LAR that were marked by prolonged suppression phases, as defined in the Methods. The following elements were selected: backward somersaults with 1 LAR (*n* = 6), 1.5 LARs (*n* = 4), or 2 LARs (*n* = 7) and forward somersaults with 1.5 LARs (*n* = 10) or 2.5 LARs (*n* = 3). The reason for the discrepancy in the number of analyzed trials of these somersaults (*n* = 37) and the number of elements evaluated in this bivariate t-test analysis (*n* = 30; see Table 2) is that artifacts during some trials prevented a precise statistical analysis of all the multiple data components without gaps.

**Figure 7:**
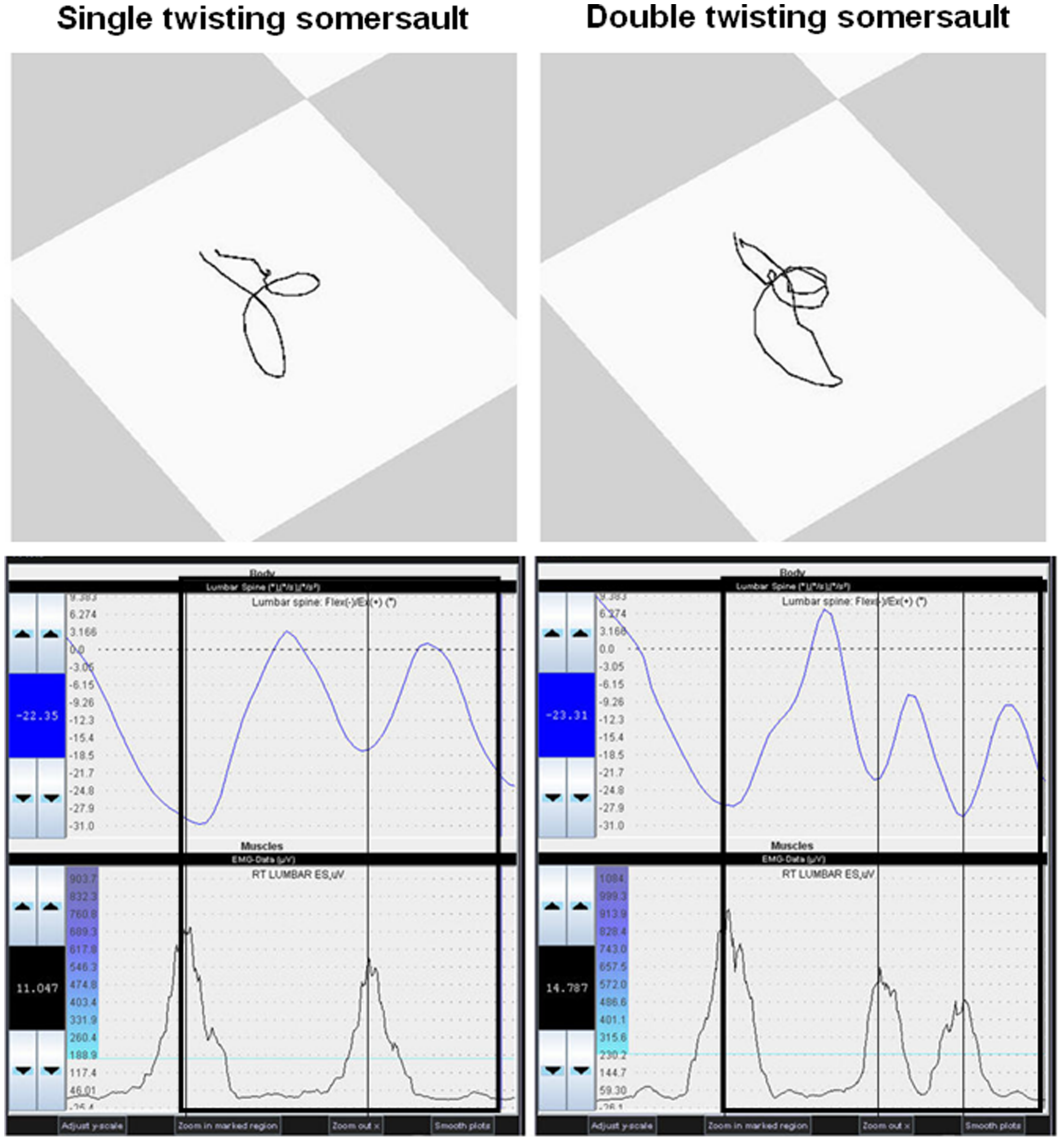
Hula movements. Examples of pelvis trajectories, lumbar spine movements and activation of the m. erector spinae during single- vs. double-twisting backward somersaults of one of the gymnasts. The trajectories of the pelvis on top of the figure are projected from a top perspective during a virtual “fixed athlete function” of the software. This function fixes the feet of the multiaxial moving model at the bottom, for better identification of the pure intersegmental movements. For a clearer identification of the “hula movement”, the pelvis trajectory line was depicted by calculating the middle between the left and the right pelvis markers (see also Video S2). The plots below show the actual motion of the lumbar spine in the sagittal plane [flexion (+); extension (−)] and the neuromuscular activation (EMG) of the m. erector spinae (named “Lumbar ES” in the plots). The thick black box marks the duration of the whole movement sequence (from the beginning to the end of the pelvis trajectory depiction). The thinner vertical lines provide a better demonstration of the temporal relationships between the plots. A detailed definition of the kinematic variables is summarized in Table 1.

The results clearly demonstrate that the athletes did not simply suspend their body movements during fast rotations, rather they moved their hips in a stereotypical hip-rotating manner (hula movement). These very small and fast intersegmental body movements were not identifiable by purely visual observation during the original multiaxial movements. At the top of Figure 7 such trajectories of the hip are exemplarily presented during a somersault with single LAR vs. double LAR (Fig. 7 and Video S2). These hip movements are closely associated with the number of LARs. For example, a backward somersault without an LAR follows the pattern of extension-flexion. Each superimposed LAR resulted in an additional hip circle, while half-LARs resulted in half hip circles. The athletes were unaware of these hula movements and unable to perceive them.

Table 4 presents results of weighted, bivariate *t*-tests of intersegmental head-body relationships during the marked suppression phases. The results show a highly significant incidence of positive correlations between the head movements and the intersegmental movements of body parts in sagittal and frontal plane (hula movements). We did not correct any temporal phase shifts between the data plots so as to avoid objections regarding manipulation of the data.

**Table 4:** Bivariate *t*-test analysis of weighted positive versus negative associations between head movements and body movements during the EREM suppression phase (*n* = 30).

Pairs of variables		*t*	*P*
**Head movements**	**Lumbar spine**		
Yaw	Lateral flection	2.312	**0.02808**
Roll	Lateral flection	5.6113	**1×10^−6^**
Pitch	Sagittal flection	3.0959	**0.004323**
**Head movements**	**Leg-trunk angle**		
Yaw	Lateral flection	3.3273	**0.002392**
Roll	Lateral flection	7.3239	**1×10^−6^**
Pitch	Sagittal flection	4.3103	**0.0001713**
**Head movements**	**Knee angle**		
Pitch	Sagittal flection	−2.2188	**0.03448**

Significant values in bold.

Table 5 summarizes the relationships between eye movements and the head and intersegmental body movements within corresponding planes during the suppression phases. As in Table 4, no correction of temporal phase shifts between the data plots was conducted. Positive associations between eye and body movements within corresponding planes were significantly more frequent than negative associations or neutral relationships. Even the knee angles showed significant relationships with vertical eye movements during the suppression phases. The reason knee angles are marked by *negative* relationships in the table (negative *t* value) is that “flexions” of the knees are defined in the *opposite direction* as “flexions” of the rest of the body joints by international convention (see also Table 1).

**Table 5:** Bivariate *t*-test of weighted positive vs. negative associations between eye movements and body movements (*n* = 30) during the EREM suppression phase.

Pairs of variables		*t*	*P*
**Eye movements**	**Head movements**		
Horizontal	Yaw axis	1.943	0.06178
Horizontal	Roll axis	1.2037	0.2385
Vertical	Pitch axis	4.7133	**0.00006**
**Eye movements**	**Lumbar spine**		
Horizontal	Lateral flection	2.5347	**0.01691**
Vertical	Sagittal flection	5.135	**0.00002**
**Eye movements**	**Leg-trunk angle**		
Horizontal	Lateral flection	2.6973	**0.01152**
Vertical	Sagittal flection	6.0924	**0.00000**
**Eye movements**	**M. erector spinae**		
Vertical	Sagittal flection	−3.8456*	**0.0006075**
**Eye movements**	**M. rectus abdom.**		
Vertical	Sagittal flection	2.547**	**0.01643**
**Eye movements**	**Knee angle**		
Vertical	Sagittal flection	−2.7715	**0.00964**

*Activation associated with downward directed eye movements;

**Activation associated with upward directed eye movements. Significant values in bold.

The eye movements were synchronized with the actual motion of the body, rather than with neuromuscular activation (EMG). This is caused by the delay between neuromuscular activation and its kinematic effect. This is best demonstrated by the finding of inverse associations between activation of the m. erector spinae and m. rectus abdominis with the movement of the eyes and the lumbar spine (Table 5). So the maximum neuromuscular activation of the back muscles is mostly synchronized during maximum ventral flexion of the lumbar spine (demonstrated in Fig. 7). This means that eye movements are *not* synchronized with neuromuscular input (EMG), but they are synchronized with its resulting kinematic output.

In contrast to the results listed in Table 5, no single significant *P* value indicated an association between eye movements and head and body movements in *non*-corresponding planes (e.g., vertical eye movements versus lateral flexion of the lumbar spine). The complete results of all the bivariate t-test analyses of the relationships between all the eye-, head- and body movements in the different planes are listed in the Table S1.

In summary, the results demonstrate a synergistic relationship between eye, head and body movements during the defined suppression phases within actively performed whole-body rotations. These relationships appear to be exact opposites of the known relationships between head and eye movements of the compensatory components of EREMs (Video S3).

## Discussion

The two most interesting findings of this study are 1) complete and prolonged suppression of nystagmus activity and other EREM functions during specific phases of the maneuvers (Figs. 3–5), and 2) the phenomenon of synergistic eye-head-body interactions during these phases (Table 5).

The main novel aspect of this phenomenon is the *functional coupling* (functional association) of eye movements with co-directed (non-compensatory) head, trunk and joint movements (including the knees). The most important remaining question is whether the functional source of this phenomenon could be reflexive, and if not, how can this phenomenon be explained.

We propose that the basis of this phenomenon is a very specific and effective combination of intentional, predictive, and reflexive mechanisms that serve one common purpose: to effectively support the requirements for directing movements. However, under normal, daily conditions, these functions are nearly completely masked by other functions. In order to support this argument, it would help to consider the main differences between the functional requirements of eye-head-body control during daily life and the very specific movement requirements investigated in this study.

With reference to human spatial orientation in daily life, two contrary functional principles exist and are permanently in conflict with each other: *stabilizing* and *pursuing/directing*. Each refers to mechanisms of gaze control and mechanisms of spinalmotor control [39], [40]. Context determines which of these functions predominates. However, during execution of any directing function, stabilizing posture and preventing falls are absolute priorities in daily life movements [40].

We believe the main difference in requirements of eye-head-body interaction during somersaults (compared to everyday movements) is the need to completely remove all environment-related mechanisms of eye, head, and body stabilization to allow effective rotational reorientation of the whole body in space (suppression phase). The required efficiency for such a complete whole-body reorientation in space would be fundamentally degraded by the activation of any stabilizing functions. The need for complete suppression of all stabilizing mechanisms, which are normally dominant in daily life, results in this pure and unmasked form of these “spinalmotor-coupled eye movements”. These requirements are never present in everyday situations.

### Functional model

**Figure 8:**
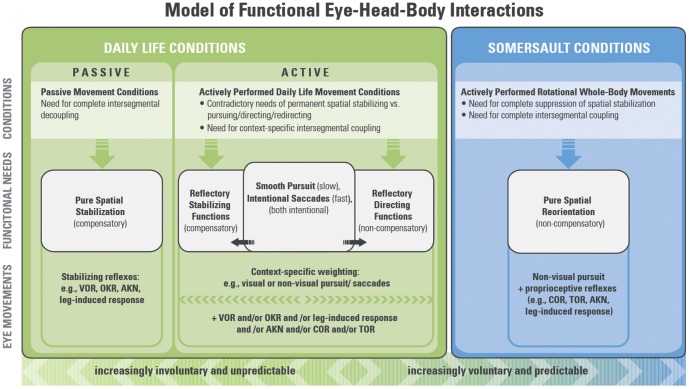
Model of eye-head-body interactions during motion. The model covers conditions from passive, completely reflexive motion, to active typical daily-life movements, to actively performed rotational whole body movements (e.g. somersault), from left to right. Vertically the corresponding description, functional needs and eye movement mechanisms are shown. See text for details.

Based on the above-mentioned considerations, we developed the following model of eye movement functions during actively performed movements (Fig. 8).

The center of Figure 8 shows requirements of actively performed movements in daily life, marked by context-specific needs with more stabilizing than pursuing/directing components. For example, to pursue a moving target, our eyes first follow the object to keep it on the fovea. For larger-amplitude object motion, the head has to be included (coupled) in this directing movement, while the trunk, pelvis, and lower extremities can remain stationary for postural stabilization. In conditions of even larger amplitudes, the trunk must also be increasingly integrated. Thus, in case of *externally driven*, *non-predictable* pursuing/directing movements, this “intersegmental coupling interface” begins at the *punctum mobile* (eyes) and moves continually to the *punctum fixum* (feet on the floor), as the amplitude increases (Video S4). However, in case of *more intentional*, *predictable*, very large or very fast directing movements, a very well coordinated, intersegmental eye-head-trunk coupling is important for producing the most effective acceleration for redirection of the body and gaze. According to the principles of predictable eye-head saccades (compare Bizzi [13]), it is necessary to begin the neuromuscular activation from the *punctum fixum* (floor), rising to the *punctum mobile* (eyes) to effectively compensate for the inertial latencies of the body segments, which are larger the closer they are to the *punctum fixum*. This “*punctum fixum - punctum mobile* principle” was already described in own measurements concerning intersegmental neuromuscular activation patterns during dynamic rotational movements around fixed axes [38]. In an additional study, it could be further demonstrated, that this “punctum fixum-punctum mobile principle” seems not only to refer to pure *spinalmotor* patterns but also include eye movements with the eyes being defined as the “punctum mobile” during fast intentional re-orienting body turns, as are common in daily life (unpublished material of a project, supported by the German Institute for Sports Science, 2012 [AZ: IIA1-070603-11]). The eyes did *not* turn *first* when being redirected to a new target most effectively (as one might expect because of their lowest inertia), in the case of *predictable* directing movements, the eyes were indeed activated *last* in order to be best synchronized with the body and head rotations (Video S5). This enables a more synchronized summing up of all the intersegmental accelerations, up to the eye movements in the head, for generating the most effective gaze shift (compare also Anastasopoulos*et al*. [17]). These results contribute an important link and a fundamental aspect within this eye-head-body interaction model, because it seems to completely explain the results during the suppression phase of the somersaults, which were also marked by this synchronization of eye movements with the actual head and body motion, but not with neuromuscular input (EMG).

We postulate that for effective motor control of any actively performed motion, independent of the context (whether directing or stabilizing dominates), voluntary gaze control (e.g. SPEM, intentional saccades) must be supported by appropriate oculomotor reflex systems. Therefore, we assume that stabilizing reflex systems also have inversely acting counterparts that serve primarily for directing movements and are mediated by intentional spinal motor coupling. For gaze stabilization, there are the compensatory reflexes (VOR and OKR). The directing functions, however, are mainly supported by COR and TOR. This would clearly explain their non-compensatory characteristics. The assumption that these reflexes play a functional role only in actively performed spinal motor movements (also speculated by Mergner *et al*. [7]) could explain why they are very weak during passive movements.

The prior findings that COR and TOR work in an non-compensatory fashion, even though the leg-induced response works mainly in a compensatory way [7], at first seem contradictory and puzzling. However, this seemingly inconsistent fact is only related to an environment-referenced perspective. From the perspective of the body's center of mass, all these proprioceptive-related reflexes work in accordance with the movement direction of the related body parts (COR with the head, TOR with the trunk, leg-induced response with legs). Thus, from this body-related point of view, the findings are, indeed, consistent. In line with this, the AKN is also known to be coupled in the direction of the arm movements [12]. The AKN obviously plays a double role in the oculomotor reflex system and serves both stabilizing and directing functions. Thus, the upper extremities seem to have quite similar functions, like the eyes, with respect to stabilizing or directing functions. During reaching or grasping movements, they move primarily synergistically with the trunk, but for stabilizing posture their movement is primarily compensatory to the trunk. Hence, the AKN mainly supports the COR and TOR during directing (reaching or grasping) tasks but supports the VOR, OKR, and leg-induced response during stabilization in daily life.

Figure 8 also depicts the functional coordination needed to effectively perform movements like somersaults, which can be described as an extreme specification of purely directing movements. They are characterized as actively performed, voluntary, planned, and highly predictable complete whole-body reorientations in space. During specific sequences (suppression phase), eye movements are driven by this unmasked synergistic co-activation of all non-compensatory spinal motor-coupled systems (e.g., COR, TOR, AKN, leg-induced response, and possibly non-visual pursuit), without being masked by stabilizing components. In Video S3, this sequence is marked by this spinal motor-related circle of eye movements, which is exactly synchronized with the intersegmental ovals of the hula movement. However, at the moment a safe landing needs to be prepared, the “system” has to be “switched back” again, because at this moment the environment-related body- and gaze-stabilizing functions need to be reactivated (supported by the known EREM functions, such as VOR, OKR and SPEM). This moment of “switching back” is marked by the change of the circular eye movement direction, as is clearly demonstrated before the landing in Video S3.

To summarize, the observations herein have resulted in the first effort to integrate the new findings within a functional model. However, the source of these “spinal motor–coupled eye movements” is not entirely clear. They may be primarily based on direct oculo-spinal interactions or could also be related to indirect serial interactions (e.g., legs to trunk to neck to eyes) or other mechanisms. Further investigation is necessary. The methods developed for this study, however, offer new insights into more functional aspects of context-specific interactions between extrinsic and intrinsic factors of eye-head-body control during complex movements. Future approaches directed at aspects of situational processes of spatial orientation and motor control could result in a broader understanding of intersegmental neuronal interactions during complex movement requirements.

## Supporting Information

Video S1 **Gaze-spot analysis.** Phases with EREM (bigger circles) and suppression phases (small circles), demonstrated by original kinematic data of a double twisting somersault in slow motion with 20 fps (50 fps represent the normal pace). For further details, see Methods and Figure 1.
(MOV)

Video S2 **Hula movement.** Original kinematic data in slow motion with 20 fps (50 fps represent the normal pace). The video demonstrates the typical hula movements during a twisting somersault. Left: Original flight curve. Right: Same trial with the athlete virtually fixed at the bottom (“fixed athlete function” of the software). The pelvis trajectory line was depicted by calculating the middle between the left and the right pelvis markers. For further details, see Methods and Figure 7.
(MOV)

Video S3 **Eye-head-body interaction during a double-twisting somersault.** Video in slow motion with 10 fps (50 fps represent the normal pace). The first part of the video sequence shows a suppression phase with “spinal motor–coupled eye movements” (the eyes circle according to the circle of the pelvis). The second part shows the change in eye movement direction before landing, according to reactivation of EREM functions. The videos content is explained by a speakers' voice.
(MOV)

Video S4 **Unpredictable directing movements.** Eye-head-body interaction during smooth pursuit of a light signal rotating slowly (but in a not predictable way) around the subject. The “intersegmental coupling interface” during this pursuing movement begins at the *punctum mobile* (eyes) and moves to *punctum fixum* (feet on the floor). The video represents the normal pace.
(MOV)

Video S5 **Predictable directing movements.** Eye-head-body interaction during predictable fast turns induced by an acoustic signal with a continuous spreading of neuromuscular activation (depicted by the colored fields in the model representing the EMG activation level) according to *punctum fixum* to *punctum mobile* activation. The first 3 movements of the video are demonstrated in normal pace with 50 fps, later the pace is reduced to 15 fps. The eyes begin not earlier to move than the first measurable head rotation (often combined with an initial eye blink). This eye-head-body interaction is demonstrated in the end of the video sequence.
(MOV)

Table S1 **All data of the bivariate t-test analysis of weighted positive and weighted negative correlations during the “suppression phases”: Alternative hypothesis (relationships between the variables) is supposed, if weighted positive and negative correlations differ significantly from 0.**
(DOC)
