# Correction: Intersegmental Eye-Head-Body Interactions during Complex Whole Body Movements

**DOI:** 10.1371/journal.pone.0112206

**Published:** 2014-10-24

**Authors:** The PLOS ONE Staff

## Notice of Republication

This article was republished on August 8, 2014, to correct errors in the author byline, citation, and references that were introduced during the production process. Please download this article again to view the correct version. The originally published, uncorrected article and the republished, corrected article are provided here for reference.

## Supporting Information

File S1 Originally published, uncorrected article.
(PDF)

File S2 Republished corrected article.
(PDF)
