# Supplementary material for: Intersegmental Eye-Head-Body Interactions during Complex Whole Body Movements
Source: PLoS One. 2014 Apr 24;9(4):e95450. doi: 10.1371/journal.pone.0095450 (PMC3998959; doi:10.1371/journal.pone.0095450)
Supplement: Table S1 — All data of the bivariate t-test analysis of weighted positive and weighted negative correlations during the “suppression phases”: Alternative hypothesis (relationships between the variables) is supposed, if weighted positive and negative correlations differ significantly from 0. (DOC) [file pone.0095450.s006.doc]

**All data of the bivariate t-test analysis of weighted positive and weighted negative correlations during the “suppression phases”: Alternative hypothesis (relationships between the variables) is supposed, if weighted positive and negative correlations differ significantly from 0.**

Left_eye._horizontal and Left_eye._vertical

One Sample t-test

data: x

t = -1.1054, df = 29, p-value = 0.2781

alternative hypothesis: true mean is not equal to 0

95 percent confidence interval:

-8.305196 2.477472

sample estimates:

mean of x

-2.913862

Left_eye._horizontal and Left_eye_to_trunk._horizontal

One Sample t-test

data: x

t = 3.6722, df = 29, p-value = 0.0009666

alternative hypothesis: true mean is not equal to 0

95 percent confidence interval:

2.648388 9.307021

sample estimates:

mean of x

5.977704

Left_eye._horizontal and Left_eye_to_trunk._vertical

One Sample t-test

data: x

t = 0.0888, df = 29, p-value = 0.9298

alternative hypothesis: true mean is not equal to 0

95 percent confidence interval:

-5.182232 5.652903

sample estimates:

mean of x

0.2353352

Left_eye._horizontal and Left_eye_to_pelvis._horizontal

One Sample t-test

t = 3.6231, df = 29, p-value = 0.001101

alternative hypothesis: true mean is not equal to 0

95 percent confidence interval:

2.286979 8.215675

sample estimates:

mean of x

5.251327

Left_eye._horizontal and Left_eye_to_pelvis._vertical

One Sample t-test

data: x

t = -0.87, df = 29, p-value = 0.3915

alternative hypothesis: true mean is not equal to 0

95 percent confidence interval:

-6.271610 2.528389

sample estimates:

mean of x

-1.871611

Left_eye._horizontal and Head_to_trunk._Pitch_axis

One Sample t-test

data: x

t = -0.5772, df = 29, p-value = 0.5682

alternative hypothesis: true mean is not equal to 0

95 percent confidence interval:

-4.293124 2.403193

sample estimates:

mean of x

-0.9449654

Left_eye._horizontal and Head_to_trunk._Roll_axis

One Sample t-test

data: x

t = 1.2037, df = 29, p-value = 0.2385

alternative hypothesis: true mean is not equal to 0

95 percent confidence interval:

-0.6142621 2.3713816

sample estimates:

mean of x

0.8785598

Left_eye._horizontal and Head_to_trunk._Yaw_axis

One Sample t-test

data: x

t = 1.943, df = 29, p-value = 0.06178

alternative hypothesis: true mean is not equal to 0

95 percent confidence interval:

-0.1341415 5.2338719

sample estimates:

mean of x

2.549865

Left_eye._horizontal and Head_to_pelvis._Pitch_axis

One Sample t-test

data: x

t = -0.8652, df = 29, p-value = 0.3941

alternative hypothesis: true mean is not equal to 0

95 percent confidence interval:

-4.676649 1.896243

sample estimates:

mean of x

-1.390203

Left_eye._horizontal and Head_to_pelvis._Roll_axis

One Sample t-test

data: x

t = 2.2018, df = 29, p-value = 0.03578

alternative hypothesis: true mean is not equal to 0

95 percent confidence interval:

0.1385813 3.7579161

sample estimates:

mean of x

1.948249

Left_eye._horizontal and Head_to_pelvis._Yaw_axis

One Sample t-test

data: x

t = 2.0296, df = 29, p-value = 0.05167

alternative hypothesis: true mean is not equal to 0

95 percent confidence interval:

-0.01907451 4.95739353

sample estimates:

mean of x

2.469160

Left_eye._horizontal and Lumbar_spine._Flex

One Sample t-test

data: x

t = -1.0366, df = 29, p-value = 0.3085

alternative hypothesis: true mean is not equal to 0

95 percent confidence interval:

-4.384952 1.435041

sample estimates:

mean of x

-1.474955

Left_eye._horizontal and Lumbar_spine._Latflex

One Sample t-test

data: x

t = 2.5347, df = 29, p-value = 0.01691

alternative hypothesis: true mean is not equal to 0

95 percent confidence interval:

0.4619477 4.3224123

sample estimates:

mean of x

2.39218

Left_eye._horizontal and Leg.trunk_left._Sagittalflex

One Sample t-test

data: x

t = -0.5572, df = 29, p-value = 0.5817

alternative hypothesis: true mean is not equal to 0

95 percent confidence interval:

-3.452103 1.973849

sample estimates:

mean of x

-0.7391273

Left_eye._horizontal and Leg.trunk_right._Sagittalflex

One Sample t-test

data: x

t = -0.6988, df = 29, p-value = 0.4903

alternative hypothesis: true mean is not equal to 0

95 percent confidence interval:

-3.861284 1.894715

sample estimates:

mean of x

-0.983284

Left_eye._horizontal and Leg.trunk_left._Lateralflex

One Sample t-test

data: x

t = 2.3982, df = 29, p-value = 0.02313

alternative hypothesis: true mean is not equal to 0

95 percent confidence interval:

0.3905675 4.9161741

sample estimates:

mean of x

2.653371

Left_eye._horizontal and Leg.trunk_right._Lateralflex

One Sample t-test

data: x

t = 2.6973, df = 29, p-value = 0.01152

alternative hypothesis: true mean is not equal to 0

95 percent confidence interval:

0.564872 4.108359

sample estimates:

mean of x

2.336616

Left_eye._horizontal and Left_knee._Flex

One Sample t-test

data: x

t = 0.5862, df = 29, p-value = 0.5623

alternative hypothesis: true mean is not equal to 0

95 percent confidence interval:

-1.060134 1.912032

sample estimates:

mean of x

0.4259491

Left_eye._horizontal and RT_PECT._MAJOR.uV

One Sample t-test

data: x

t = 0.3905, df = 29, p-value = 0.699

alternative hypothesis: true mean is not equal to 0

95 percent confidence interval:

-1.586471 2.335214

sample estimates:

mean of x

0.3743715

Left_eye._horizontal and RT_RECT.ABDOM.LO..uV

One Sample t-test

data: x

t = 1.001, df = 29, p-value = 0.3251

alternative hypothesis: true mean is not equal to 0

95 percent confidence interval:

-2.267352 6.614707

sample estimates:

mean of x

2.173677

Left_eye._horizontal and RT_RECTUS_FEM..uV

One Sample t-test

data: x

t = -0.877, df = 29, p-value = 0.3877

alternative hypothesis: true mean is not equal to 0

95 percent confidence interval:

-2.0168755 0.8063243

sample estimates:

mean of x

-0.6052756

Left_eye._horizontal and RT_POST.DELTOID.uV

One Sample t-test

data: x

t = -0.4707, df = 29, p-value = 0.6414

alternative hypothesis: true mean is not equal to 0

95 percent confidence interval:

-1.744734 1.091898

sample estimates:

mean of x

-0.3264182

Left_eye._horizontal and RT_LUMBAR_ES.uV

One Sample t-test

data: x

t = -0.7813, df = 29, p-value = 0.441

alternative hypothesis: true mean is not equal to 0

95 percent confidence interval:

-6.513017 2.912498

sample estimates:

mean of x

-1.800259

Left_eye._horizontal and LT_BICEPS_FEM..uV

One Sample t-test

data: x

t = 1.8117, df = 29, p-value = 0.0804

alternative hypothesis: true mean is not equal to 0

95 percent confidence interval:

-0.3777795 6.2401539

sample estimates:

mean of x

2.931187

Left_eye._vertical and Left_eye_to_trunk._horizontal

One Sample t-test

data: x

t = -0.3564, df = 29, p-value = 0.7241

alternative hypothesis: true mean is not equal to 0

95 percent confidence interval:

-4.334283 3.047974

sample estimates:

mean of x

-0.6431545

Left_eye._vertical and Left_eye_to_trunk._vertical

One Sample t-test

data: x

t = 5.9094, df = 29, p-value = 2.043e-06

alternative hypothesis: true mean is not equal to 0

95 percent confidence interval:

7.846594 16.152679

sample estimates:

mean of x

11.99964

Left_eye._vertical and Left_eye_to_pelvis._horizontal

One Sample t-test

data: x

t = 0.0101, df = 29, p-value = 0.992

alternative hypothesis: true mean is not equal to 0

95 percent confidence interval:

-3.552653 3.588032

sample estimates:

mean of x

0.01768934

Left_eye._vertical and Left_eye_to_pelvis._vertical

One Sample t-test

data: x

t = 7.3411, df = 29, p-value = 4.361e-08

alternative hypothesis: true mean is not equal to 0

95 percent confidence interval:

6.682893 11.844642

sample estimates:

mean of x

9.263767

Left_eye._vertical and Head_to_trunk._Pitch_axis

One Sample t-test

data: x

t = 4.7133, df = 29, p-value = 5.624e-05

alternative hypothesis: true mean is not equal to 0

95 percent confidence interval:

1.593045 4.035327

sample estimates:

mean of x

2.814186

Left_eye._vertical and Head_to_trunk._Roll_axis

One Sample t-test

data: x

t = 0.5005, df = 29, p-value = 0.6205

alternative hypothesis: true mean is not equal to 0

95 percent confidence interval:

-1.181911 1.947756

sample estimates:

mean of x

0.3829223

Left_eye._vertical and Head_to_trunk._Yaw_axis

One Sample t-test

data: x

t = 0.0564, df = 29, p-value = 0.9554

alternative hypothesis: true mean is not equal to 0

95 percent confidence interval:

-3.104148 3.280327

sample estimates:

mean of x

0.0880895

Left_eye._vertical and Head_to_pelvis._Pitch_axis

One Sample t-test

data: x

t = 6.4206, df = 29, p-value = 5.059e-07

alternative hypothesis: true mean is not equal to 0

95 percent confidence interval:

2.574914 4.982166

sample estimates:

mean of x

3.77854

Left_eye._vertical and Head_to_pelvis._Roll_axis

One Sample t-test

data: x

t = 0.8385, df = 29, p-value = 0.4086

alternative hypothesis: true mean is not equal to 0

95 percent confidence interval:

-0.9678457 2.3128665

sample estimates:

mean of x

0.6725104

Left_eye._vertical and Head_to_pelvis._Yaw_axis

One Sample t-test

data: x

t = 0.3491, df = 29, p-value = 0.7295

alternative hypothesis: true mean is not equal to 0

95 percent confidence interval:

-2.618621 3.696673

sample estimates:

mean of x

0.5390259

Left_eye._vertical and Lumbar_spine._Flex

One Sample t-test

data: x

t = 5.135, df = 29, p-value = 1.743e-05

alternative hypothesis: true mean is not equal to 0

95 percent confidence interval:

2.137050 4.966229

sample estimates:

mean of x

3.551639

Left_eye._vertical and Lumbar_spine._Latflex

One Sample t-test

data: x

t = 1.684, df = 29, p-value = 0.1029

alternative hypothesis: true mean is not equal to 0

95 percent confidence interval:

-0.3697005 3.8166720

sample estimates:

mean of x

1.723486

Left_eye._vertical and Leg.trunk_left._Sagittalflex

One Sample t-test

data: x

t = 5.8716, df = 29, p-value = 2.267e-06

alternative hypothesis: true mean is not equal to 0

95 percent confidence interval:

4.285570 8.866972

sample estimates:

mean of x

6.576271

Left_eye._vertical and Leg.trunk_right._Sagittalflex

One Sample t-test

data: x

t = 6.0924, df = 29, p-value = 1.236e-06

alternative hypothesis: true mean is not equal to 0

95 percent confidence interval:

5.473446 11.005410

sample estimates:

mean of x

8.239428

Left_eye._vertical and Leg.trunk_left._Lateralflex

One Sample t-test

data: x

t = 1.2224, df = 29, p-value = 0.2314

alternative hypothesis: true mean is not equal to 0

95 percent confidence interval:

-1.033855 4.105562

sample estimates:

mean of x

1.535854

Left_eye._vertical and Leg.trunk_right._Lateralflex

One Sample t-test

data: x

t = 1.9325, df = 29, p-value = 0.06312

alternative hypothesis: true mean is not equal to 0

95 percent confidence interval:

-0.1194600 4.2133319

sample estimates:

mean of x

2.046936

Left_eye._vertical and Left_knee._Flex

One Sample t-test

data: x

t = -2.7715, df = 29, p-value = 0.00964

alternative hypothesis: true mean is not equal to 0

95 percent confidence interval:

-7.106369 -1.071550

sample estimates:

mean of x

-4.08896

Left_eye._vertical and RT_PECT._MAJOR.uV

One Sample t-test

data: x

t = 3.4796, df = 29, p-value = 0.001608

alternative hypothesis: true mean is not equal to 0

95 percent confidence interval:

1.945774 7.494466

sample estimates:

mean of x

4.72012

Left_eye._vertical and RT_RECT.ABDOM.LO..uV

One Sample t-test

data: x

t = 2.547, df = 29, p-value = 0.01643

alternative hypothesis: true mean is not equal to 0

95 percent confidence interval:

0.4233243 3.8740348

sample estimates:

mean of x

2.148680

Left_eye._vertical and RT_RECTUS_FEM..uV

One Sample t-test

data: x

t = -5.2859, df = 29, p-value = 1.146e-05

alternative hypothesis: true mean is not equal to 0

95 percent confidence interval:

-4.130522 -1.825858

sample estimates:

mean of x

-2.97819

Left_eye._vertical and RT_POST.DELTOID.uV

One Sample t-test

data: x

t = -6.1358, df = 29, p-value = 1.098e-06

alternative hypothesis: true mean is not equal to 0

95 percent confidence interval:

-4.906345 -2.453217

sample estimates:

mean of x

-3.679781

Left_eye._vertical and RT_LUMBAR_ES.uV

One Sample t-test

data: x

t = -3.8456, df = 29, p-value = 0.0006075

alternative hypothesis: true mean is not equal to 0

95 percent confidence interval:

-8.994406 -2.748871

sample estimates:

mean of x

-5.871639

Left_eye._vertical and LT_BICEPS_FEM..uV

One Sample t-test

data: x

t = -1.0047, df = 29, p-value = 0.3234

alternative hypothesis: true mean is not equal to 0

95 percent confidence interval:

-5.819646 1.985487

sample estimates:

mean of x

-1.917080

Left_eye_to_trunk._horizontal and Left_eye_to_trunk._vertical

One Sample t-test

data: x

t = 0.3228, df = 29, p-value = 0.7491

alternative hypothesis: true mean is not equal to 0

95 percent confidence interval:

-1.884164 2.590499

sample estimates:

mean of x

0.3531676

Left_eye_to_trunk._horizontal and Left_eye_to_pelvis._horizontal

One Sample t-test

data: x

t = 3.8727, df = 29, p-value = 0.0005646

alternative hypothesis: true mean is not equal to 0

95 percent confidence interval:

24.82318 80.38493

sample estimates:

mean of x

52.60405

Left_eye_to_trunk._horizontal and Left_eye_to_pelvis._vertical

One Sample t-test

data: x

t = -1.7557, df = 29, p-value = 0.0897

alternative hypothesis: true mean is not equal to 0

95 percent confidence interval:

-3.0451890 0.2319675

sample estimates:

mean of x

-1.406611

Left_eye_to_trunk._horizontal and Head_to_trunk._Pitch_axis

One Sample t-test

data: x

t = 0.7613, df = 29, p-value = 0.4526

alternative hypothesis: true mean is not equal to 0

95 percent confidence interval:

-1.016082 2.221007

sample estimates:

mean of x

0.6024625

Left_eye_to_trunk._horizontal and Head_to_trunk._Roll_axis

One Sample t-test

data: x

t = 4.9924, df = 29, p-value = 2.591e-05

alternative hypothesis: true mean is not equal to 0

95 percent confidence interval:

4.701028 11.225701

sample estimates:

mean of x

7.963364

Left_eye_to_trunk._horizontal and Head_to_trunk._Yaw_axis

One Sample t-test

data: x

t = 4.2227, df = 29, p-value = 0.0002179

alternative hypothesis: true mean is not equal to 0

95 percent confidence interval:

20.59800 59.29282

sample estimates:

mean of x

39.94541

Left_eye_to_trunk._horizontal and Head_to_pelvis._Pitch_axis

One Sample t-test

data: x

t = -1.1957, df = 29, p-value = 0.2415

alternative hypothesis: true mean is not equal to 0

95 percent confidence interval:

-2.5227328 0.6612645

sample estimates:

mean of x

-0.9307342

Left_eye_to_trunk._horizontal and Head_to_pelvis._Roll_axis

One Sample t-test

data: x

t = 3.2453, df = 29, p-value = 0.002955

alternative hypothesis: true mean is not equal to 0

95 percent confidence interval:

1.549780 6.832234

sample estimates:

mean of x

4.191007

Left_eye_to_trunk._horizontal and Head_to_pelvis._Yaw_axis

One Sample t-test

data: x

t = 3.6429, df = 29, p-value = 0.001045

alternative hypothesis: true mean is not equal to 0

95 percent confidence interval:

13.19058 46.96182

sample estimates:

mean of x

30.0762

Left_eye_to_trunk._horizontal and Lumbar_spine._Flex

One Sample t-test

data: x

t = -0.8978, df = 29, p-value = 0.3767

alternative hypothesis: true mean is not equal to 0

95 percent confidence interval:

-2.4096364 0.9395122

sample estimates:

mean of x

-0.7350621

Left_eye_to_trunk._horizontal and Lumbar_spine._Latflex

One Sample t-test

data: x

t = 2.3427, df = 29, p-value = 0.02622

alternative hypothesis: true mean is not equal to 0

95 percent confidence interval:

0.3983076 5.8752895

sample estimates:

mean of x

3.136799

Left_eye_to_trunk._horizontal and Leg.trunk_left._Sagittalflex

One Sample t-test

data: x

t = -1.4157, df = 29, p-value = 0.1675

alternative hypothesis: true mean is not equal to 0

95 percent confidence interval:

-2.6287357 0.4781517

sample estimates:

mean of x

-1.075292

Left_eye_to_trunk._horizontal and Leg.trunk_right._Sagittalflex

One Sample t-test

data: x

t = -1.7544, df = 29, p-value = 0.08993

alternative hypothesis: true mean is not equal to 0

95 percent confidence interval:

-2.9095307 0.2227379

sample estimates:

mean of x

-1.343396

Left_eye_to_trunk._horizontal and Leg.trunk_left._Lateralflex

One Sample t-test

data: x

t = -0.7278, df = 29, p-value = 0.4726

alternative hypothesis: true mean is not equal to 0

95 percent confidence interval:

-43.09509 20.47362

sample estimates:

mean of x

-11.31074

Left_eye_to_trunk._horizontal and Leg.trunk_right._Lateralflex

One Sample t-test

data: x

t = 2.2845, df = 29, p-value = 0.02985

alternative hypothesis: true mean is not equal to 0

95 percent confidence interval:

0.4262519 7.7128307

sample estimates:

mean of x

4.069541

Left_eye_to_trunk._horizontal and Left_knee._Flex

One Sample t-test

data: x

t = 0.9978, df = 29, p-value = 0.3266

alternative hypothesis: true mean is not equal to 0

95 percent confidence interval:

-1.604159 4.660327

sample estimates:

mean of x

1.528084

Left_eye_to_trunk._horizontal and RT_PECT._MAJOR.uV

One Sample t-test

data: x

t = -0.5025, df = 29, p-value = 0.6191

alternative hypothesis: true mean is not equal to 0

95 percent confidence interval:

-4.000086 2.422174

sample estimates:

mean of x

-0.7889558

Left_eye_to_trunk._horizontal and RT_RECT.ABDOM.LO..uV

One Sample t-test

data: x

t = 0.027, df = 29, p-value = 0.9786

alternative hypothesis: true mean is not equal to 0

95 percent confidence interval:

-1.393243 1.430570

sample estimates:

mean of x

0.01866338

Left_eye_to_trunk._horizontal and RT_RECTUS_FEM..uV

One Sample t-test

data: x

t = 1.3618, df = 29, p-value = 0.1838

alternative hypothesis: true mean is not equal to 0

95 percent confidence interval:

-0.714346 3.560911

sample estimates:

mean of x

1.423282

Left_eye_to_trunk._horizontal and RT_POST.DELTOID.uV

One Sample t-test

data: x

t = 1.6337, df = 29, p-value = 0.1131

alternative hypothesis: true mean is not equal to 0

95 percent confidence interval:

-0.371222 3.318606

sample estimates:

mean of x

1.473692

Left_eye_to_trunk._horizontal and RT_LUMBAR_ES.uV

One Sample t-test

data: x

t = 0.7814, df = 29, p-value = 0.4409

alternative hypothesis: true mean is not equal to 0

95 percent confidence interval:

-1.918254 4.290460

sample estimates:

mean of x

1.186103

Left_eye_to_trunk._horizontal and LT_BICEPS_FEM..uV

One Sample t-test

data: x

t = 1.6826, df = 29, p-value = 0.1032

alternative hypothesis: true mean is not equal to 0

95 percent confidence interval:

-0.66067 6.79083

sample estimates:

mean of x

3.06508

Left_eye_to_trunk._vertical and Left_eye_to_pelvis._horizontal

One Sample t-test

data: x

t = 0.8223, df = 29, p-value = 0.4176

alternative hypothesis: true mean is not equal to 0

95 percent confidence interval:

-1.331977 3.123314

sample estimates:

mean of x

0.8956684

Left_eye_to_trunk._vertical and Left_eye_to_pelvis._vertical

One Sample t-test

data: x

t = 5.3542, df = 29, p-value = 9.48e-06

alternative hypothesis: true mean is not equal to 0

95 percent confidence interval:

11.75681 26.29034

sample estimates:

mean of x

19.02357

Left_eye_to_trunk._vertical and Head_to_trunk._Pitch_axis

One Sample t-test

data: x

t = 6.1603, df = 29, p-value = 1.027e-06

alternative hypothesis: true mean is not equal to 0

95 percent confidence interval:

9.33460 18.61344

sample estimates:

mean of x

13.97402

Left_eye_to_trunk._vertical and Head_to_trunk._Roll_axis

One Sample t-test

data: x

t = 1.0698, df = 29, p-value = 0.2935

alternative hypothesis: true mean is not equal to 0

95 percent confidence interval:

-0.6391118 2.0410734

sample estimates:

mean of x

0.7009808

Left_eye_to_trunk._vertical and Head_to_trunk._Yaw_axis

One Sample t-test

data: x

t = 0.7319, df = 29, p-value = 0.4701

alternative hypothesis: true mean is not equal to 0

95 percent confidence interval:

-1.356464 2.868348

sample estimates:

mean of x

0.755942

Left_eye_to_trunk._vertical and Head_to_pelvis._Pitch_axis

One Sample t-test

data: x

t = 6.3308, df = 29, p-value = 6.454e-07

alternative hypothesis: true mean is not equal to 0

95 percent confidence interval:

6.310057 12.332861

sample estimates:

mean of x

9.32146

Left_eye_to_trunk._vertical and Head_to_pelvis._Roll_axis

One Sample t-test

data: x

t = 0.2158, df = 29, p-value = 0.8307

alternative hypothesis: true mean is not equal to 0

95 percent confidence interval:

-1.626642 2.010383

sample estimates:

mean of x

0.1918703

Left_eye_to_trunk._vertical and Head_to_pelvis._Yaw_axis

One Sample t-test

data: x

t = 1.1895, df = 29, p-value = 0.2439

alternative hypothesis: true mean is not equal to 0

95 percent confidence interval:

-0.8974816 3.3927895

sample estimates:

mean of x

1.247654

Left_eye_to_trunk._vertical and Lumbar_spine._Flex

One Sample t-test

data: x

t = 2.9011, df = 29, p-value = 0.007028

alternative hypothesis: true mean is not equal to 0

95 percent confidence interval:

1.225915 7.085169

sample estimates:

mean of x

4.155542

Left_eye_to_trunk._vertical and Lumbar_spine._Latflex

One Sample t-test

data: x

t = 0.9922, df = 29, p-value = 0.3293

alternative hypothesis: true mean is not equal to 0

95 percent confidence interval:

-1.006323 2.902841

sample estimates:

mean of x

0.948259

Left_eye_to_trunk._vertical and Leg.trunk_left._Sagittalflex

One Sample t-test

data: x

t = 4.0429, df = 29, p-value = 0.000356

alternative hypothesis: true mean is not equal to 0

95 percent confidence interval:

3.52068 10.72959

sample estimates:

mean of x

7.125134

Left_eye_to_trunk._vertical and Leg.trunk_right._Sagittalflex

One Sample t-test

data: x

t = 3.7565, df = 29, p-value = 0.0007716

alternative hypothesis: true mean is not equal to 0

95 percent confidence interval:

3.068886 10.404298

sample estimates:

mean of x

6.736592

Left_eye_to_trunk._vertical and Leg.trunk_left._Lateralflex

One Sample t-test

data: x

t = 0.4187, df = 29, p-value = 0.6785

alternative hypothesis: true mean is not equal to 0

95 percent confidence interval:

-1.829343 2.771294

sample estimates:

mean of x

0.4709757

Left_eye_to_trunk._vertical and Leg.trunk_right._Lateralflex

One Sample t-test

data: x

t = 1.0964, df = 29, p-value = 0.2819

alternative hypothesis: true mean is not equal to 0

95 percent confidence interval:

-0.7462307 2.4707338

sample estimates:

mean of x

0.8622516

Left_eye_to_trunk._vertical and Left_knee._Flex

One Sample t-test

data: x

t = -4.3224, df = 29, p-value = 0.0001657

alternative hypothesis: true mean is not equal to 0

95 percent confidence interval:

-6.230953 -2.228303

sample estimates:

mean of x

-4.229628

Left_eye_to_trunk._vertical and RT_PECT._MAJOR.uV One Sample t-test

data: x

t = 2.969, df = 29, p-value = 0.00594

alternative hypothesis: true mean is not equal to 0

95 percent confidence interval:

1.256413 6.819745

sample estimates:

mean of x

4.038079

Left_eye_to_trunk._vertical and RT_RECT.ABDOM.LO..uV

One Sample t-test

data: x

t = 2.7545, df = 29, p-value = 0.01005

alternative hypothesis: true mean is not equal to 0

95 percent confidence interval:

0.6457506 4.3700791

sample estimates:

mean of x

2.507915

Left_eye_to_trunk._vertical and RT_RECTUS_FEM..uV

One Sample t-test

data: x

t = -4.5594, df = 29, p-value = 8.616e-05

alternative hypothesis: true mean is not equal to 0

95 percent confidence interval:

-4.299041 -1.636504

sample estimates:

mean of x

-2.967772

Left_eye_to_trunk._vertical and RT_POST.DELTOID.uV

One Sample t-test

data: x

t = -5.7775, df = 29, p-value = 2.938e-06

alternative hypothesis: true mean is not equal to 0

95 percent confidence interval:

-4.941046 -2.357397

sample estimates:

mean of x

-3.649221

Left_eye_to_trunk._vertical and RT_LUMBAR_ES.uV

One Sample t-test

data: x

t = -2.8332, df = 29, p-value = 0.0083

alternative hypothesis: true mean is not equal to 0

95 percent confidence interval:

-8.605114 -1.389904

sample estimates:

mean of x

-4.997509

Left_eye_to_trunk._vertical and LT_BICEPS_FEM..uV

One Sample t-test

data: x

t = -1.4251, df = 29, p-value = 0.1648

alternative hypothesis: true mean is not equal to 0

95 percent confidence interval:

-5.650022 1.009574

sample estimates:

mean of x

-2.320224

Left_eye_to_pelvis._horizontal and Left_eye_to_pelvis._vertical

One Sample t-test

data: x

t = -0.8548, df = 29, p-value = 0.3997

alternative hypothesis: true mean is not equal to 0

95 percent confidence interval:

-2.2432812 0.9208778

sample estimates:

mean of x

-0.6612017

Left_eye_to_pelvis._horizontal and Head_to_trunk._Pitch_axis

One Sample t-test

data: x

t = 1.1658, df = 29, p-value = 0.2532

alternative hypothesis: true mean is not equal to 0

95 percent confidence interval:

-0.6432812 2.3486430

sample estimates:

mean of x

0.8526809

Left_eye_to_pelvis._horizontal and Head_to_trunk._Roll_axis

One Sample t-test

data: x

t = 5.5734, df = 29, p-value = 5.163e-06

alternative hypothesis: true mean is not equal to 0

95 percent confidence interval:

5.828644 12.586245

sample estimates:

mean of x

9.207444

Left_eye_to_pelvis._horizontal and Head_to_trunk._Yaw_axis

One Sample t-test

data: x

t = 3.5135, df = 29, p-value = 0.001471

alternative hypothesis: true mean is not equal to 0

95 percent confidence interval:

11.81222 44.72051

sample estimates:

mean of x

28.26637

Left_eye_to_pelvis._horizontal and Head_to_pelvis._Pitch_axis

One Sample t-test

data: x

t = -0.6011, df = 29, p-value = 0.5524

alternative hypothesis: true mean is not equal to 0

95 percent confidence interval:

-2.039174 1.112749

sample estimates:

mean of x

-0.4632126

Left_eye_to_pelvis._horizontal and Head_to_pelvis._Roll_axis

One Sample t-test

data: x

t = 4.4526, df = 29, p-value = 0.0001157

alternative hypothesis: true mean is not equal to 0

95 percent confidence interval:

3.473097 9.374343

sample estimates:

mean of x

6.42372

Left_eye_to_pelvis._horizontal and Head_to_pelvis._Yaw_axis

One Sample t-test

data: x

t = 4.1831, df = 29, p-value = 0.0002428

alternative hypothesis: true mean is not equal to 0

95 percent confidence interval:

23.38873 68.13821

sample estimates:

mean of x

45.76347

Left_eye_to_pelvis._horizontal and Lumbar_spine._Flex

One Sample t-test

data: x

t = -0.8631, df = 29, p-value = 0.3952

alternative hypothesis: true mean is not equal to 0

95 percent confidence interval:

-2.3029094 0.9360886

sample estimates:

mean of x

-0.6834104

Left_eye_to_pelvis._horizontal and Lumbar_spine._Latflex

One Sample t-test

data: x

t = 3.6148, df = 29, p-value = 0.001126

alternative hypothesis: true mean is not equal to 0

95 percent confidence interval:

2.470708 8.909787

sample estimates:

mean of x

5.690247

Left_eye_to_pelvis._horizontal and Leg.trunk_left._Sagittalflex

One Sample t-test

data: x

t = -0.9408, df = 29, p-value = 0.3546

alternative hypothesis: true mean is not equal to 0

95 percent confidence interval:

-2.2605745 0.8361343

sample estimates:

mean of x

-0.7122201

Left_eye_to_pelvis._horizontal and Leg.trunk_right._Sagittalflex

One Sample t-test

data: x

t = -1.3989, df = 29, p-value = 0.1725

alternative hypothesis: true mean is not equal to 0

95 percent confidence interval:

-2.5261549 0.4740869

sample estimates:

mean of x

-1.026034

Left_eye_to_pelvis._horizontal and Leg.trunk_left._Lateralflex

One Sample t-test

data: x

t = 1.2405, df = 29, p-value = 0.2247

alternative hypothesis: true mean is not equal to 0

95 percent confidence interval:

-2.629119 10.735535

sample estimates:

mean of x

4.053208

Left_eye_to_pelvis._horizontal and Leg.trunk_right._Lateralflex

One Sample t-test

data: x

t = 2.8563, df = 29, p-value = 0.007844

alternative hypothesis: true mean is not equal to 0

95 percent confidence interval:

1.528133 9.234531

sample estimates:

mean of x

5.381332

Left_eye_to_pelvis._horizontal and Left_knee._Flex

One Sample t-test

data: x

t = 0.7316, df = 29, p-value = 0.4703

alternative hypothesis: true mean is not equal to 0

95 percent confidence interval:

-2.112015 4.464425

sample estimates:

mean of x

1.176205

Left_eye_to_pelvis._horizontal and RT_PECT._MAJOR.uV

One Sample t-test

data: x

t = -0.4566, df = 29, p-value = 0.6514

alternative hypothesis: true mean is not equal to 0

95 percent confidence interval:

-4.146709 2.633188

sample estimates:

mean of x

-0.7567606

Left_eye_to_pelvis._horizontal and RT_RECT.ABDOM.LO..uV

One Sample t-test

data: x

t = 0.3263, df = 29, p-value = 0.7466

alternative hypothesis: true mean is not equal to 0

95 percent confidence interval:

-1.405624 1.939217

sample estimates:

mean of x

0.2667961

Left_eye_to_pelvis._horizontal and RT_RECTUS_FEM..uV

One Sample t-test

data: x

t = 1.072, df = 29, p-value = 0.2926

alternative hypothesis: true mean is not equal to 0

95 percent confidence interval:

-1.043916 3.343508

sample estimates:

mean of x

1.149796

Left_eye_to_pelvis._horizontal and RT_POST.DELTOID.uV

One Sample t-test

data: x

t = 1.0656, df = 29, p-value = 0.2954

alternative hypothesis: true mean is not equal to 0

95 percent confidence interval:

-0.9497183 3.0157550

sample estimates:

mean of x

1.033018

Left_eye_to_pelvis._horizontal and RT_LUMBAR_ES.uV

One Sample t-test

data: x

t = 0.7067, df = 29, p-value = 0.4854

alternative hypothesis: true mean is not equal to 0

95 percent confidence interval:

-2.002252 4.116301

sample estimates:

mean of x

1.057025

Left_eye_to_pelvis._horizontal and LT_BICEPS_FEM..uV

One Sample t-test

data: x

t = 1.7318, df = 29, p-value = 0.09393

alternative hypothesis: true mean is not equal to 0

95 percent confidence interval:

-0.5602791 6.7520100

sample estimates:

mean of x

3.095865

Left_eye_to_pelvis._vertical and Head_to_trunk._Pitch_axis

One Sample t-test

data: x

t = 4.6236, df = 29, p-value = 7.213e-05

alternative hypothesis: true mean is not equal to 0

95 percent confidence interval:

5.395289 13.954741

sample estimates:

mean of x

9.675015

Left_eye_to_pelvis._vertical and Head_to_trunk._Roll_axis

One Sample t-test

data: x

t = 0.296, df = 29, p-value = 0.7694

alternative hypothesis: true mean is not equal to 0

95 percent confidence interval:

-0.7699124 1.0304601

sample estimates:

mean of x

0.1302739

Left_eye_to_pelvis._vertical and Head_to_trunk._Yaw_axis

One Sample t-test

data: x

t = -0.7881, df = 29, p-value = 0.437

alternative hypothesis: true mean is not equal to 0

95 percent confidence interval:

-2.0245660 0.8982839

sample estimates:

mean of x

-0.563141

Left_eye_to_pelvis._vertical and Head_to_pelvis._Pitch_axis

One Sample t-test

data: x

t = 6.7658, df = 29, p-value = 1.996e-07

alternative hypothesis: true mean is not equal to 0

95 percent confidence interval:

15.83891 29.56348

sample estimates:

mean of x

22.70119

Left_eye_to_pelvis._vertical and Head_to_pelvis._Roll_axis

One Sample t-test

data: x

t = 0.1148, df = 29, p-value = 0.9094

alternative hypothesis: true mean is not equal to 0

95 percent confidence interval:

-1.997961 2.235682

sample estimates:

mean of x

0.1188603

Left_eye_to_pelvis._vertical and Head_to_pelvis._Yaw_axis

One Sample t-test

data: x

t = 0.1349, df = 29, p-value = 0.8936

alternative hypothesis: true mean is not equal to 0

95 percent confidence interval:

-1.484922 1.694674

sample estimates:

mean of x

0.1048758

Left_eye_to_pelvis._vertical and Lumbar_spine._Flex

One Sample t-test

data: x

t = 3.995, df = 29, p-value = 0.0004055

alternative hypothesis: true mean is not equal to 0

95 percent confidence interval:

5.129386 15.890383

sample estimates:

mean of x

10.50988

Left_eye_to_pelvis._vertical and Lumbar_spine._Latflex

One Sample t-test

data: x

t = 0.8751, df = 29, p-value = 0.3887

alternative hypothesis: true mean is not equal to 0

95 percent confidence interval:

-1.016061 2.535762

sample estimates:

mean of x

0.7598505

Left_eye_to_pelvis._vertical and Leg.trunk_left._Sagittalflex

One Sample t-test

data: x

t = 4.8363, df = 29, p-value = 3.998e-05

alternative hypothesis: true mean is not equal to 0

95 percent confidence interval:

8.330132 20.538323

sample estimates:

mean of x

14.43423

Left_eye_to_pelvis._vertical and Leg.trunk_right._Sagittalflex

One Sample t-test

data: x

t = 4.4474, df = 29, p-value = 0.0001174

alternative hypothesis: true mean is not equal to 0

95 percent confidence interval:

7.630674 20.624185

sample estimates:

mean of x

14.12743

Left_eye_to_pelvis._vertical and Leg.trunk_left._Lateralflex

One Sample t-test

data: x

t = 0.462, df = 29, p-value = 0.6475

alternative hypothesis: true mean is not equal to 0

95 percent confidence interval:

-1.784088 2.825339

sample estimates:

mean of x

0.5206253

Left_eye_to_pelvis._vertical and Leg.trunk_right._Lateralflex

One Sample t-test

data: x

t = 1.4226, df = 29, p-value = 0.1655

alternative hypothesis: true mean is not equal to 0

95 percent confidence interval:

-0.465052 2.590087

sample estimates:

mean of x

1.062517

Left_eye_to_pelvis._vertical and Left_knee._Flex

One Sample t-test

data: x

t = -3.0163, df = 29, p-value = 0.00528

alternative hypothesis: true mean is not equal to 0

95 percent confidence interval:

-6.783172 -1.301326

sample estimates:

mean of x

-4.042249

Left_eye_to_pelvis._vertical and RT_PECT._MAJOR.uV

One Sample t-test

data: x

t = 2.9764, df = 29, p-value = 0.005832

alternative hypothesis: true mean is not equal to 0

95 percent confidence interval:

1.681820 9.069692

sample estimates:

mean of x

5.375756

Left_eye_to_pelvis._vertical and RT_RECT.ABDOM.LO..uV

One Sample t-test

data: x

t = 2.8462, df = 29, p-value = 0.00804

alternative hypothesis: true mean is not equal to 0

95 percent confidence interval:

0.4068542 2.4845512

sample estimates:

mean of x

1.445703

Left_eye_to_pelvis._vertical and RT_RECTUS_FEM..uV

One Sample t-test

data: x

t = -5.1295, df = 29, p-value = 1.77e-05

alternative hypothesis: true mean is not equal to 0

95 percent confidence interval:

-3.784784 -1.626988

sample estimates:

mean of x

-2.705886

Left_eye_to_pelvis._vertical and RT_POST.DELTOID.uV

One Sample t-test

data: x

t = -6.7085, df = 29, p-value = 2.328e-07

alternative hypothesis: true mean is not equal to 0

95 percent confidence interval:

-5.123921 -2.729615

sample estimates:

mean of x

-3.926768

Left_eye_to_pelvis._vertical and RT_LUMBAR_ES.uV

One Sample t-test

data: x

t = -4.1757, df = 29, p-value = 0.0002478

alternative hypothesis: true mean is not equal to 0

95 percent confidence interval:

-8.019053 -2.746297

sample estimates:

mean of x

-5.382675

Left_eye_to_pelvis._vertical and LT_BICEPS_FEM..uV

One Sample t-test

data: x

t = -1.6507, df = 29, p-value = 0.1096

alternative hypothesis: true mean is not equal to 0

95 percent confidence interval:

-5.3439484 0.5704667

sample estimates:

mean of x

-2.386741

Head_to_trunk._Pitch_axis and Head_to_trunk._Roll_axis

One Sample t-test

data: x

t = 1.5849, df = 29, p-value = 0.1238

alternative hypothesis: true mean is not equal to 0

95 percent confidence interval:

-0.4518675 3.5636426

sample estimates:

mean of x

1.555888

Head_to_trunk._Pitch_axis and Head_to_trunk._Yaw_axis

One Sample t-test

data: x

t = 1.6363, df = 29, p-value = 0.1126

alternative hypothesis: true mean is not equal to 0

95 percent confidence interval:

-1.035860 9.326026

sample estimates:

mean of x

4.145083

Head_to_trunk._Pitch_axis and Head_to_pelvis._Pitch_axis

One Sample t-test

data: x

t = 4.0567, df = 29, p-value = 0.0003429

alternative hypothesis: true mean is not equal to 0

95 percent confidence interval:

6.204568 18.822199

sample estimates:

mean of x

12.51338

Head_to_trunk._Pitch_axis and Head_to_pelvis._Roll_axis

One Sample t-test

data: x

t = 1.0558, df = 29, p-value = 0.2998

alternative hypothesis: true mean is not equal to 0

95 percent confidence interval:

-1.468421 4.601966

sample estimates:

mean of x

1.566772

Head_to_trunk._Pitch_axis and Head_to_pelvis._Yaw_axis

One Sample t-test

data: x

t = 1.7942, df = 29, p-value = 0.08322

alternative hypothesis: true mean is not equal to 0

95 percent confidence interval:

-0.342946 5.244219

sample estimates:

mean of x

2.450637

Head_to_trunk._Pitch_axis and Lumbar_spine._Flex

One Sample t-test

data: x

t = 3.0959, df = 29, p-value = 0.004323

alternative hypothesis: true mean is not equal to 0

95 percent confidence interval:

1.006540 4.925221

sample estimates:

mean of x

2.965880

Head_to_trunk._Pitch_axis and Lumbar_spine._Latflex

One Sample t-test

data: x

t = 0.7226, df = 29, p-value = 0.4757

alternative hypothesis: true mean is not equal to 0

95 percent confidence interval:

-1.278846 2.676325

sample estimates:

mean of x

0.6987398

Head_to_trunk._Pitch_axis and Leg.trunk_left._Sagittalflex

One Sample t-test

data: x

t = 4.3103, df = 29, p-value = 0.0001713

alternative hypothesis: true mean is not equal to 0

95 percent confidence interval:

1.743591 4.892276

sample estimates:

mean of x

3.317934

Head_to_trunk._Pitch_axis and Leg.trunk_right._Sagittalflex

One Sample t-test

data: x

t = 4.3635, df = 29, p-value = 0.0001480

alternative hypothesis: true mean is not equal to 0

95 percent confidence interval:

1.645240 4.548127

sample estimates:

mean of x

3.096683

Head_to_trunk._Pitch_axis and Leg.trunk_left._Lateralflex

One Sample t-test

data: x

t = 1.4876, df = 29, p-value = 0.1476

alternative hypothesis: true mean is not equal to 0

95 percent confidence interval:

-0.5829938 3.6936450

sample estimates:

mean of x

1.555326

Head_to_trunk._Pitch_axis and Leg.trunk_right._Lateralflex

One Sample t-test

data: x

t = 1.0125, df = 29, p-value = 0.3197

alternative hypothesis: true mean is not equal to 0

95 percent confidence interval:

-0.6694029 1.9820684

sample estimates:

mean of x

0.6563328

Head_to_trunk._Pitch_axis and Left_knee._Flex

One Sample t-test

data: x

t = -2.2188, df = 29, p-value = 0.03448

alternative hypothesis: true mean is not equal to 0

95 percent confidence interval:

-4.8509694 -0.1974714

sample estimates:

mean of x

-2.524220

Head_to_trunk._Pitch_axis and RT_PECT._MAJOR.uV

One Sample t-test

data: x

t = 2.2821, df = 29, p-value = 0.03001

alternative hypothesis: true mean is not equal to 0

95 percent confidence interval:

0.2090655 3.8195934

sample estimates:

mean of x

2.014329

Head_to_trunk._Pitch_axis and RT_RECT.ABDOM.LO..uV

One Sample t-test

data: x

t = -0.2685, df = 29, p-value = 0.7902

alternative hypothesis: true mean is not equal to 0

95 percent confidence interval:

-3.329727 2.556819

sample estimates:

mean of x

-0.3864544

Head_to_trunk._Pitch_axis and RT_RECTUS_FEM..uV

One Sample t-test

data: x

t = -1.9579, df = 29, p-value = 0.05993

alternative hypothesis: true mean is not equal to 0

95 percent confidence interval:

-3.19415484 0.06971211

sample estimates:

mean of x

-1.562221

Head_to_trunk._Pitch_axis and RT_POST.DELTOID.uV

One Sample t-test

data: x

t = -3.1608, df = 29, p-value = 0.003667

alternative hypothesis: true mean is not equal to 0

95 percent confidence interval:

-4.2503353 -0.9107736

sample estimates:

mean of x

-2.580554

Head_to_trunk._Pitch_axis and RT_LUMBAR_ES.uV

One Sample t-test

data: x

t = -0.4678, df = 29, p-value = 0.6435

alternative hypothesis: true mean is not equal to 0

95 percent confidence interval:

-1.618415 1.015921

sample estimates:

mean of x

-0.3012471

Head_to_trunk._Pitch_axis and LT_BICEPS_FEM..uV

One Sample t-test

data: x

t = 0.7842, df = 29, p-value = 0.4393

alternative hypothesis: true mean is not equal to 0

95 percent confidence interval:

-2.813999 6.313856

sample estimates:

mean of x

1.749928

Head_to_trunk._Roll_axis and Head_to_trunk._Yaw_axis

One Sample t-test

data: x

t = 3.4193, df = 29, p-value = 0.001883

alternative hypothesis: true mean is not equal to 0

95 percent confidence interval:

4.100314 16.306748

sample estimates:

mean of x

10.20353

Head_to_trunk._Roll_axis and Head_to_pelvis._Pitch_axis

One Sample t-test

data: x

t = 1.4824, df = 29, p-value = 0.1490

alternative hypothesis: true mean is not equal to 0

95 percent confidence interval:

-0.3040278 1.9054978

sample estimates:

mean of x

0.800735

Head_to_trunk._Roll_axis and Head_to_pelvis._Roll_axis

One Sample t-test

data: x

t = 5.6035, df = 29, p-value = 4.751e-06

alternative hypothesis: true mean is not equal to 0

95 percent confidence interval:

5.724103 12.304345

sample estimates:

mean of x

9.014224

Head_to_trunk._Roll_axis and Head_to_pelvis._Yaw_axis

One Sample t-test

data: x

t = 5.0807, df = 29, p-value = 2.027e-05

alternative hypothesis: true mean is not equal to 0

95 percent confidence interval:

6.00890 14.10629

sample estimates:

mean of x

10.05759

Head_to_trunk._Roll_axis and Lumbar_spine._Flex

One Sample t-test

data: x

t = 0.2933, df = 29, p-value = 0.7714

alternative hypothesis: true mean is not equal to 0

95 percent confidence interval:

-1.204823 1.608247

sample estimates:

mean of x

0.2017118

Head_to_trunk._Roll_axis and Lumbar_spine._Latflex

One Sample t-test

data: x

t = 5.6113, df = 29, p-value = 4.650e-06

alternative hypothesis: true mean is not equal to 0

95 percent confidence interval:

2.219394 4.765180

sample estimates:

mean of x

3.492287

Head_to_trunk._Roll_axis and Leg.trunk_left._Sagittalflex

One Sample t-test

data: x

t = -0.5029, df = 29, p-value = 0.6189

alternative hypothesis: true mean is not equal to 0

95 percent confidence interval:

-1.2876139 0.7793904

sample estimates:

mean of x

-0.2541117

Head_to_trunk._Roll_axis and Leg.trunk_right._Sagittalflex

One Sample t-test

data: x

t = -0.8782, df = 29, p-value = 0.3871

alternative hypothesis: true mean is not equal to 0

95 percent confidence interval:

-1.4426302 0.5759076

sample estimates:

mean of x

-0.4333613

Head_to_trunk._Roll_axis and Leg.trunk_left._Lateralflex

One Sample t-test

data: x

t = 5.3465, df = 29, p-value = 9.686e-06

alternative hypothesis: true mean is not equal to 0

95 percent confidence interval:

2.572017 5.758929

sample estimates:

mean of x

4.165473

Head_to_trunk._Roll_axis and Leg.trunk_right._Lateralflex

One Sample t-test

data: x

t = 7.3239, df = 29, p-value = 4.562e-08

alternative hypothesis: true mean is not equal to 0

95 percent confidence interval:

2.706941 4.804576

sample estimates:

mean of x

3.755759

Head_to_trunk._Roll_axis and Left_knee._Flex

One Sample t-test

data: x

t = -0.4273, df = 29, p-value = 0.6723

alternative hypothesis: true mean is not equal to 0

95 percent confidence interval:

-2.585238 1.691663

sample estimates:

mean of x

-0.4467878

Head_to_trunk._Roll_axis and RT_PECT._MAJOR.uV

One Sample t-test

data: x

t = -1.0605, df = 29, p-value = 0.2977

alternative hypothesis: true mean is not equal to 0

95 percent confidence interval:

-2.8404970 0.9006536

sample estimates:

mean of x

-0.9699217

Head_to_trunk._Roll_axis and RT_RECT.ABDOM.LO..uV

One Sample t-test

data: x

t = 0.0793, df = 29, p-value = 0.9374

alternative hypothesis: true mean is not equal to 0

95 percent confidence interval:

-1.826201 1.973440

sample estimates:

mean of x

0.07361983

Head_to_trunk._Roll_axis and RT_RECTUS_FEM..uV

One Sample t-test

data: x

t = -0.3573, df = 29, p-value = 0.7235

alternative hypothesis: true mean is not equal to 0

95 percent confidence interval:

-2.796275 1.964649

sample estimates:

mean of x

-0.4158128

Head_to_trunk._Roll_axis and RT_POST.DELTOID.uV

One Sample t-test

data: x

t = -0.5829, df = 29, p-value = 0.5645

alternative hypothesis: true mean is not equal to 0

95 percent confidence interval:

-1.766426 0.982900

sample estimates:

mean of x

-0.3917630

Head_to_trunk._Roll_axis and RT_LUMBAR_ES.uV

One Sample t-test

data: x

t = 1.4556, df = 29, p-value = 0.1562

alternative hypothesis: true mean is not equal to 0

95 percent confidence interval:

-0.3602137 2.1388798

sample estimates:

mean of x

0.889333

Head_to_trunk._Roll_axis and LT_BICEPS_FEM..uV

One Sample t-test

data: x

t = -0.3203, df = 29, p-value = 0.751

alternative hypothesis: true mean is not equal to 0

95 percent confidence interval:

-1.415061 1.031887

sample estimates:

mean of x

-0.1915872

Head_to_trunk._Yaw_axis and Head_to_pelvis._Pitch_axis

One Sample t-test

data: x

t = 0.5787, df = 29, p-value = 0.5673

alternative hypothesis: true mean is not equal to 0

95 percent confidence interval:

-2.025268 3.623566

sample estimates:

mean of x

0.7991491

Head_to_trunk._Yaw_axis and Head_to_pelvis._Roll_axis

One Sample t-test

data: x

t = 4.8763, df = 29, p-value = 3.577e-05

alternative hypothesis: true mean is not equal to 0

95 percent confidence interval:

2.168805 5.302406

sample estimates:

mean of x

3.735606

Head_to_trunk._Yaw_axis and Head_to_pelvis._Yaw_axis

One Sample t-test

data: x

t = 3.9383, df = 29, p-value = 0.0004729

alternative hypothesis: true mean is not equal to 0

95 percent confidence interval:

17.07259 53.96228

sample estimates:

mean of x

35.51743

Head_to_trunk._Yaw_axis and Lumbar_spine._Flex

One Sample t-test

data: x

t = -0.3749, df = 29, p-value = 0.7104

alternative hypothesis: true mean is not equal to 0

95 percent confidence interval:

-1.908843 1.317391

sample estimates:

mean of x

-0.2957260

Head_to_trunk._Yaw_axis and Lumbar_spine._Latflex

One Sample t-test

data: x

t = 2.312, df = 29, p-value = 0.02808

alternative hypothesis: true mean is not equal to 0

95 percent confidence interval:

0.2463388 4.0236597

sample estimates:

mean of x

2.134999

Head_to_trunk._Yaw_axis and Leg.trunk_left._Sagittalflex

One Sample t-test

data: x

t = -0.0669, df = 29, p-value = 0.9471

alternative hypothesis: true mean is not equal to 0

95 percent confidence interval:

-2.957456 2.770171

sample estimates:

mean of x

-0.0936424

Head_to_trunk._Yaw_axis and Leg.trunk_right._Sagittalflex

One Sample t-test

data: x

t = -0.0585, df = 29, p-value = 0.9538

alternative hypothesis: true mean is not equal to 0

95 percent confidence interval:

-3.322116 3.137440

sample estimates:

mean of x

-0.09233815

Head_to_trunk._Yaw_axis and Leg.trunk_left._Lateralflex

One Sample t-test

data: x

t = 3.3273, df = 29, p-value = 0.002392

alternative hypothesis: true mean is not equal to 0

95 percent confidence interval:

1.107631 4.641455

sample estimates:

mean of x

2.874543

Head_to_trunk._Yaw_axis and Leg.trunk_right._Lateralflex

One Sample t-test

data: x

t = 2.5439, df = 29, p-value = 0.01655

alternative hypothesis: true mean is not equal to 0

95 percent confidence interval:

0.4470109 4.1136929

sample estimates:

mean of x

2.280352

Head_to_trunk._Yaw_axis and Left_knee._Flex

One Sample t-test

data: x

t = -1.0184, df = 29, p-value = 0.3169

alternative hypothesis: true mean is not equal to 0

95 percent confidence interval:

-4.584351 1.536512

sample estimates:

mean of x

-1.523919

Head_to_trunk._Yaw_axis and RT_PECT._MAJOR.uV

One Sample t-test

data: x

t = -0.181, df = 29, p-value = 0.8576

alternative hypothesis: true mean is not equal to 0

95 percent confidence interval:

-2.254890 1.888252

sample estimates:

mean of x

-0.183319

Head_to_trunk._Yaw_axis and RT_RECT.ABDOM.LO..uV

One Sample t-test

data: x

t = -0.353, df = 29, p-value = 0.7267

alternative hypothesis: true mean is not equal to 0

95 percent confidence interval:

-1.905055 1.344283

sample estimates:

mean of x

-0.2803856

Head_to_trunk._Yaw_axis and RT_RECTUS_FEM..uV

One Sample t-test

data: x

t = -0.3558, df = 29, p-value = 0.7245

alternative hypothesis: true mean is not equal to 0

95 percent confidence interval:

-2.631635 1.851648

sample estimates:

mean of x

-0.3899938

Head_to_trunk._Yaw_axis and RT_POST.DELTOID.uV

One Sample t-test

data: x

t = -0.5757, df = 29, p-value = 0.5693

alternative hypothesis: true mean is not equal to 0

95 percent confidence interval:

-2.523884 1.415139

sample estimates:

mean of x

-0.5543721

Head_to_trunk._Yaw_axis and RT_LUMBAR_ES.uV

One Sample t-test

data: x

t = -0.1864, df = 29, p-value = 0.8535

alternative hypothesis: true mean is not equal to 0

95 percent confidence interval:

-3.890787 3.240929

sample estimates:

mean of x

-0.3249288

Head_to_trunk._Yaw_axis and LT_BICEPS_FEM..uV

One Sample t-test

data: x

t = 0.5984, df = 29, p-value = 0.5542

alternative hypothesis: true mean is not equal to 0

95 percent confidence interval:

-1.119153 2.044851

sample estimates:

mean of x

0.4628488

Head_to_pelvis._Pitch_axis and Head_to_pelvis._Roll_axis

One Sample t-test

data: x

t = 1.0746, df = 29, p-value = 0.2914

alternative hypothesis: true mean is not equal to 0

95 percent confidence interval:

-1.687242 5.423273

sample estimates:

mean of x

1.868015

Head_to_pelvis._Pitch_axis and Head_to_pelvis._Yaw_axis

One Sample t-test

data: x

t = 1.1392, df = 29, p-value = 0.2639

alternative hypothesis: true mean is not equal to 0

95 percent confidence interval:

-1.767553 6.212610

sample estimates:

mean of x

2.222528

Head_to_pelvis._Pitch_axis and Lumbar_spine._Flex

One Sample t-test

data: x

t = 7.171, df = 29, p-value = 6.812e-08

alternative hypothesis: true mean is not equal to 0

95 percent confidence interval:

7.08683 12.74227

sample estimates:

mean of x

9.914549

Head_to_pelvis._Pitch_axis and Lumbar_spine._Latflex

One Sample t-test

data: x

t = 0.5796, df = 29, p-value = 0.5667

alternative hypothesis: true mean is not equal to 0

95 percent confidence interval:

-1.523781 2.728855

sample estimates:

mean of x

0.602537

Head_to_pelvis._Pitch_axis and Leg.trunk_left._Sagittalflex

One Sample t-test

data: x

t = 7.8579, df = 29, p-value = 1.149e-08

alternative hypothesis: true mean is not equal to 0

95 percent confidence interval:

7.234634 12.325762

sample estimates:

mean of x

9.780198

Head_to_pelvis._Pitch_axis and Leg.trunk_right._Sagittalflex

One Sample t-test

data: x

t = 8.6723, df = 29, p-value = 1.505e-09

alternative hypothesis: true mean is not equal to 0

95 percent confidence interval:

7.124962 11.522706

sample estimates:

mean of x

9.323834

Head_to_pelvis._Pitch_axis and Leg.trunk_left._Lateralflex

One Sample t-test

data: x

t = 1.0514, df = 29, p-value = 0.3018

alternative hypothesis: true mean is not equal to 0

95 percent confidence interval:

-1.039202 3.237812

sample estimates:

mean of x

1.099305

Head_to_pelvis._Pitch_axis and Leg.trunk_right._Lateralflex

One Sample t-test

data: x

t = 0.7796, df = 29, p-value = 0.4419

alternative hypothesis: true mean is not equal to 0

95 percent confidence interval:

-0.7304432 1.6303454

sample estimates:

mean of x

0.4499511

Head_to_pelvis._Pitch_axis and Left_knee._Flex

One Sample t-test

data: x

t = -1.927, df = 29, p-value = 0.06383

alternative hypothesis: true mean is not equal to 0

95 percent confidence interval:

-4.1504135 0.1235257

sample estimates:

mean of x

-2.013444

Head_to_pelvis._Pitch_axis and RT_PECT._MAJOR.uV

One Sample t-test

data: x

t = 3.0516, df = 29, p-value = 0.004833

alternative hypothesis: true mean is not equal to 0

95 percent confidence interval:

1.267306 6.418346

sample estimates:

mean of x

3.842826

Head_to_pelvis._Pitch_axis and RT_RECT.ABDOM.LO..uV

One Sample t-test

data: x

t = -0.2047, df = 29, p-value = 0.8393

alternative hypothesis: true mean is not equal to 0

95 percent confidence interval:

-3.942928 3.225595

sample estimates:

mean of x

-0.3586666

Head_to_pelvis._Pitch_axis and RT_RECTUS_FEM..uV

One Sample t-test

data: x

t = -2.3559, df = 29, p-value = 0.02545

alternative hypothesis: true mean is not equal to 0

95 percent confidence interval:

-2.8561519 -0.2016149

sample estimates:

mean of x

-1.528883

Head_to_pelvis._Pitch_axis and RT_POST.DELTOID.uV

One Sample t-test

data: x

t = -3.2452, df = 29, p-value = 0.002956

alternative hypothesis: true mean is not equal to 0

95 percent confidence interval:

-5.328452 -1.208568

sample estimates:

mean of x

-3.268510

Head_to_pelvis._Pitch_axis and RT_LUMBAR_ES.uV

One Sample t-test

data: x

t = -2.7873, df = 29, p-value = 0.00928

alternative hypothesis: true mean is not equal to 0

95 percent confidence interval:

-4.1062925 -0.6305213

sample estimates:

mean of x

-2.368407

Head_to_pelvis._Pitch_axis and LT_BICEPS_FEM..uV

One Sample t-test

data: x

t = 0.6301, df = 29, p-value = 0.5336

alternative hypothesis: true mean is not equal to 0

95 percent confidence interval:

-3.175993 6.004393

sample estimates:

mean of x

1.414200

Head_to_pelvis._Roll_axis and Head_to_pelvis._Yaw_axis

One Sample t-test

data: x

t = 4.5836, df = 29, p-value = 8.057e-05

alternative hypothesis: true mean is not equal to 0

95 percent confidence interval:

3.796011 9.913065

sample estimates:

mean of x

6.854538

Head_to_pelvis._Roll_axis and Lumbar_spine._Flex

One Sample t-test

data: x

t = 0.1724, df = 29, p-value = 0.8643

alternative hypothesis: true mean is not equal to 0

95 percent confidence interval:

-2.323612 2.751393

sample estimates:

mean of x

0.2138904

Head_to_pelvis._Roll_axis and Lumbar_spine._Latflex

One Sample t-test

data: x

t = 5.8459, df = 29, p-value = 2.433e-06

alternative hypothesis: true mean is not equal to 0

95 percent confidence interval:

6.64312 13.79280

sample estimates:

mean of x

10.21796

Head_to_pelvis._Roll_axis and Leg.trunk_left._Sagittalflex

One Sample t-test

data: x

t = 0.0529, df = 29, p-value = 0.9582

alternative hypothesis: true mean is not equal to 0

95 percent confidence interval:

-2.359359 2.484593

sample estimates:

mean of x

0.06261725

Head_to_pelvis._Roll_axis and Leg.trunk_right._Sagittalflex

One Sample t-test

data: x

t = 0.0597, df = 29, p-value = 0.9528

alternative hypothesis: true mean is not equal to 0

95 percent confidence interval:

-2.363116 2.505223

sample estimates:

mean of x

0.0710534

Head_to_pelvis._Roll_axis and Leg.trunk_left._Lateralflex

One Sample t-test

data: x

t = 5.6359, df = 29, p-value = 4.343e-06

alternative hypothesis: true mean is not equal to 0

95 percent confidence interval:

5.874553 12.566752

sample estimates:

mean of x

9.220653

Head_to_pelvis._Roll_axis and Leg.trunk_right._Lateralflex

One Sample t-test

data: x

t = 6.6049, df = 29, p-value = 3.075e-07

alternative hypothesis: true mean is not equal to 0

95 percent confidence interval:

6.420932 12.181098

sample estimates:

mean of x

9.301015

Head_to_pelvis._Roll_axis and Left_knee._Flex

One Sample t-test

data: x

t = -0.187, df = 29, p-value = 0.853

alternative hypothesis: true mean is not equal to 0

95 percent confidence interval:

-2.035017 1.694010

sample estimates:

mean of x

-0.1705036

Head_to_pelvis._Roll_axis and RT_PECT._MAJOR.uV

One Sample t-test

data: x

t = -0.4463, df = 29, p-value = 0.6587

alternative hypothesis: true mean is not equal to 0

95 percent confidence interval:

-3.040008 1.950955

sample estimates:

mean of x

-0.5445262

Head_to_pelvis._Roll_axis and RT_RECT.ABDOM.LO..uV

One Sample t-test

data: x

t = 0.0783, df = 29, p-value = 0.9381

alternative hypothesis: true mean is not equal to 0

95 percent confidence interval:

-2.097695 2.264772

sample estimates:

mean of x

0.08353844

Head_to_pelvis._Roll_axis and RT_RECTUS_FEM..uV

One Sample t-test

data: x

t = -0.171, df = 29, p-value = 0.8654

alternative hypothesis: true mean is not equal to 0

95 percent confidence interval:

-2.556264 2.161852

sample estimates:

mean of x

-0.1972062

Head_to_pelvis._Roll_axis and RT_POST.DELTOID.uV

One Sample t-test

data: x

t = -1.1735, df = 29, p-value = 0.2502

alternative hypothesis: true mean is not equal to 0

95 percent confidence interval:

-3.6832213 0.9975565

sample estimates:

mean of x

-1.342832

Head_to_pelvis._Roll_axis and RT_LUMBAR_ES.uV

One Sample t-test

data: x

t = 0.2567, df = 29, p-value = 0.7992

alternative hypothesis: true mean is not equal to 0

95 percent confidence interval:

-1.532336 1.972277

sample estimates:

mean of x

0.2199700

Head_to_pelvis._Roll_axis and LT_BICEPS_FEM..uV

One Sample t-test

data: x

t = 1.7649, df = 29, p-value = 0.0881

alternative hypothesis: true mean is not equal to 0

95 percent confidence interval:

-0.2780215 3.7791214

sample estimates:

mean of x

1.75055

Head_to_pelvis._Yaw_axis and Lumbar_spine._Latflex

One Sample t-test

data: x

t = 3.8423, df = 29, p-value = 0.0006128

alternative hypothesis: true mean is not equal to 0

95 percent confidence interval:

2.604271 8.531911

sample estimates:

mean of x

5.568091

Head_to_pelvis._Yaw_axis and Leg.trunk_left._Sagittalflex

One Sample t-test

data: x

t = 0.0694, df = 29, p-value = 0.9452

alternative hypothesis: true mean is not equal to 0

95 percent confidence interval:

-2.415036 2.584643

sample estimates:

mean of x

0.08480329

Head_to_pelvis._Yaw_axis and Leg.trunk_right._Sagittalflex

One Sample t-test

data: x

t = 0.0644, df = 29, p-value = 0.9491

alternative hypothesis: true mean is not equal to 0

95 percent confidence interval:

-2.729327 2.906656

sample estimates:

mean of x

0.08866463

Head_to_pelvis._Yaw_axis and Leg.trunk_left._Lateralflex

One Sample t-test

data: x

t = 3.9978, df = 29, p-value = 0.0004025

alternative hypothesis: true mean is not equal to 0

95 percent confidence interval:

3.061916 9.476408

sample estimates:

mean of x

6.269162

Head_to_pelvis._Yaw_axis and Leg.trunk_right._Lateralflex

One Sample t-test

data: x

t = 3.8294, df = 29, p-value = 0.0006345

alternative hypothesis: true mean is not equal to 0

95 percent confidence interval:

2.390112 7.869724

sample estimates:

mean of x

5.129918

Head_to_pelvis._Yaw_axis and Left_knee._Flex

One Sample t-test

data: x

t = -1.149, df = 29, p-value = 0.2599

alternative hypothesis: true mean is not equal to 0

95 percent confidence interval:

-4.716883 1.323451

sample estimates:

mean of x

-1.696716

Head_to_pelvis._Yaw_axis and RT_PECT._MAJOR.uV

One Sample t-test

data: x

t = 0.0639, df = 29, p-value = 0.9495

alternative hypothesis: true mean is not equal to 0

95 percent confidence interval:

-1.786035 1.901239

sample estimates:

mean of x

0.05760227

Head_to_pelvis._Yaw_axis and RT_RECT.ABDOM.LO..uV

One Sample t-test

data: x

t = 0.4056, df = 29, p-value = 0.688

alternative hypothesis: true mean is not equal to 0

95 percent confidence interval:

-2.382870 3.561850

sample estimates:

mean of x

0.5894902

Head_to_pelvis._Yaw_axis and RT_RECTUS_FEM..uV

One Sample t-test

data: x

t = -0.5391, df = 29, p-value = 0.5939

alternative hypothesis: true mean is not equal to 0

95 percent confidence interval:

-3.566257 2.078316

sample estimates:

mean of x

-0.7439708

Head_to_pelvis._Yaw_axis and RT_POST.DELTOID.uV

One Sample t-test

data: x

t = -1.0156, df = 29, p-value = 0.3182

alternative hypothesis: true mean is not equal to 0

95 percent confidence interval:

-3.452899 1.161484

sample estimates:

mean of x

-1.145708

Head_to_pelvis._Yaw_axis and RT_LUMBAR_ES.uV

One Sample t-test

data: x

t = -0.2751, df = 29, p-value = 0.7852

alternative hypothesis: true mean is not equal to 0

95 percent confidence interval:

-3.718742 2.836988

sample estimates:

mean of x

-0.4408768

Head_to_pelvis._Yaw_axis and LT_BICEPS_FEM..uV

One Sample t-test

data: x

t = 2.5496, df = 29, p-value = 0.01633

alternative hypothesis: true mean is not equal to 0

95 percent confidence interval:

0.2773731 2.5268830

sample estimates:

mean of x

1.402128

Lumbar_spine._Flex and Lumbar_spine._Latflex

One Sample t-test

data: x

t = -0.0122, df = 29, p-value = 0.9904

alternative hypothesis: true mean is not equal to 0

95 percent confidence interval:

-1.516271 1.498344

sample estimates:

mean of x

-0.008963716

Lumbar_spine._Flex and Leg.trunk_left._Sagittalflex

One Sample t-test

data: x

t = 7.7455, df = 29, p-value = 1.532e-08

alternative hypothesis: true mean is not equal to 0

95 percent confidence interval:

9.809277 16.848360

sample estimates:

mean of x

13.32882

Lumbar_spine._Flex and Leg.trunk_right._Sagittalflex

One Sample t-test

data: x

t = 6.8754, df = 29, p-value = 1.49e-07

alternative hypothesis: true mean is not equal to 0

95 percent confidence interval:

9.347972 17.264388

sample estimates:

mean of x

13.30618

Lumbar_spine._Flex and Leg.trunk_left._Lateralflex

One Sample t-test

data: x

t = -0.6837, df = 29, p-value = 0.4996

alternative hypothesis: true mean is not equal to 0

95 percent confidence interval:

-2.306463 1.150805

sample estimates:

mean of x

-0.5778293

Lumbar_spine._Flex and Leg.trunk_right._Lateralflex

One Sample t-test

data: x

t = 0.406, df = 29, p-value = 0.6877

alternative hypothesis: true mean is not equal to 0

95 percent confidence interval:

-1.410879 2.109721

sample estimates:

mean of x

0.3494209

Lumbar_spine._Flex and Left_knee._Flex

One Sample t-test

data: x

t = -1.1413, df = 29, p-value = 0.2631

alternative hypothesis: true mean is not equal to 0

95 percent confidence interval:

-1.8726475 0.5312417

sample estimates:

mean of x

-0.6707029

Lumbar_spine._Flex and RT_PECT._MAJOR.uV

One Sample t-test

data: x

t = 3.9276, df = 29, p-value = 0.0004868

alternative hypothesis: true mean is not equal to 0

95 percent confidence interval:

1.719615 5.456331

sample estimates:

mean of x

3.587973

Lumbar_spine._Flex and RT_RECT.ABDOM.LO..uV

One Sample t-test

data: x

t = -0.0863, df = 29, p-value = 0.9318

alternative hypothesis: true mean is not equal to 0

95 percent confidence interval:

-3.403612 3.128090

sample estimates:

mean of x

-0.1377611

Lumbar_spine._Flex and RT_RECTUS_FEM..uV

One Sample t-test

data: x

t = -0.786, df = 29, p-value = 0.4382

alternative hypothesis: true mean is not equal to 0

95 percent confidence interval:

-1.2870067 0.5723876

sample estimates:

mean of x

-0.3573095

Lumbar_spine._Flex and RT_POST.DELTOID.uV

One Sample t-test

data: x

t = -3.1163, df = 29, p-value = 0.004105

alternative hypothesis: true mean is not equal to 0

95 percent confidence interval:

-3.8398327 -0.7968308

sample estimates:

mean of x

-2.318332

Lumbar_spine._Flex and RT_LUMBAR_ES.uV

One Sample t-test

data: x

t = -3.7211, df = 29, p-value = 0.0008484

alternative hypothesis: true mean is not equal to 0

95 percent confidence interval:

-4.299019 -1.249402

sample estimates:

mean of x

-2.774211

Lumbar_spine._Flex and LT_BICEPS_FEM..uV

One Sample t-test

data: x

t = -1.3554, df = 29, p-value = 0.1857

alternative hypothesis: true mean is not equal to 0

95 percent confidence interval:

-3.3383732 0.6771687

sample estimates:

mean of x

-1.330602

Lumbar_spine._Latflex and Leg.trunk_left._Sagittalflex

One Sample t-test

data: x

t = 0.062, df = 29, p-value = 0.951

alternative hypothesis: true mean is not equal to 0

95 percent confidence interval:

-2.161885 2.297104

sample estimates:

mean of x

0.06760933

Lumbar_spine._Latflex and Leg.trunk_right._Sagittalflex

One Sample t-test

data: x

t = 0.0899, df = 29, p-value = 0.929

alternative hypothesis: true mean is not equal to 0

95 percent confidence interval:

-2.068616 2.258748

sample estimates:

mean of x

0.09506583

Lumbar_spine._Latflex and Leg.trunk_left._Lateralflex

One Sample t-test

data: x

t = 4.8112, df = 29, p-value = 4.287e-05

alternative hypothesis: true mean is not equal to 0

95 percent confidence interval:

10.24283 25.39063

sample estimates:

mean of x

17.81673

Lumbar_spine._Latflex and Leg.trunk_right._Lateralflex

One Sample t-test

data: x

t = 5.849, df = 29, p-value = 2.412e-06

alternative hypothesis: true mean is not equal to 0

95 percent confidence interval:

12.13919 25.19344

sample estimates:

mean of x

18.66632

Lumbar_spine._Latflex and Left_knee._Flex

One Sample t-test

data: x

t = 0.3526, df = 29, p-value = 0.727

alternative hypothesis: true mean is not equal to 0

95 percent confidence interval:

-2.004170 2.839056

sample estimates:

mean of x

0.4174431

Lumbar_spine._Latflex and RT_PECT._MAJOR.uV

One Sample t-test

data: x

t = 0.5464, df = 29, p-value = 0.589

alternative hypothesis: true mean is not equal to 0

95 percent confidence interval:

-1.233594 2.133025

sample estimates:

mean of x

0.4497152

Lumbar_spine._Latflex and RT_RECT.ABDOM.LO..uV

One Sample t-test

data: x

t = 0.481, df = 29, p-value = 0.6342

alternative hypothesis: true mean is not equal to 0

95 percent confidence interval:

-2.653170 4.284701

sample estimates:

mean of x

0.8157655

Lumbar_spine._Latflex and RT_RECTUS_FEM..uV

One Sample t-test

data: x

t = -0.2114, df = 29, p-value = 0.834

alternative hypothesis: true mean is not equal to 0

95 percent confidence interval:

-2.171878 1.764942

sample estimates:

mean of x

-0.2034681

Lumbar_spine._Latflex and RT_POST.DELTOID.uV

One Sample t-test

data: x

t = -1.2774, df = 29, p-value = 0.2116

alternative hypothesis: true mean is not equal to 0

95 percent confidence interval:

-4.608350 1.064944

sample estimates:

mean of x

-1.771703

Lumbar_spine._Latflex and RT_LUMBAR_ES.uV

One Sample t-test

data: x

t = -0.6674, df = 29, p-value = 0.5098

alternative hypothesis: true mean is not equal to 0

95 percent confidence interval:

-3.336089 1.694529

sample estimates:

mean of x

-0.82078

Lumbar_spine._Latflex and LT_BICEPS_FEM..uV

One Sample t-test

data: x

t = 2.1601, df = 29, p-value = 0.03917

alternative hypothesis: true mean is not equal to 0

95 percent confidence interval:

0.09032014 3.30638225

sample estimates:

mean of x

1.698351

Leg.trunk_left._Sagittalflex and Leg.trunk_right._Sagittalflex

One Sample t-test

data: x

t = 5.0032, df = 29, p-value = 2.514e-05

alternative hypothesis: true mean is not equal to 0

95 percent confidence interval:

46.03066 109.68553

sample estimates:

mean of x

77.85809

Leg.trunk_left._Sagittalflex and Leg.trunk_left._Lateralflex

One Sample t-test

data: x

t = -0.7985, df = 29, p-value = 0.4311

alternative hypothesis: true mean is not equal to 0

95 percent confidence interval:

-2.532698 1.110362

sample estimates:

mean of x

-0.7111676

Leg.trunk_left._Sagittalflex and Leg.trunk_right._Lateralflex

One Sample t-test

data: x

t = 0.3184, df = 29, p-value = 0.7525

alternative hypothesis: true mean is not equal to 0

95 percent confidence interval:

-1.796821 2.459441

sample estimates:

mean of x

0.33131

Leg.trunk_left._Sagittalflex and Left_knee._Flex

One Sample t-test

data: x

t = -1.8567, df = 29, p-value = 0.07355

alternative hypothesis: true mean is not equal to 0

95 percent confidence interval:

-6.8248465 0.3298374

sample estimates:

mean of x

-3.247505

Leg.trunk_left._Sagittalflex and RT_PECT._MAJOR.uV

One Sample t-test

data: x

t = 3.4079, df = 29, p-value = 0.001940

alternative hypothesis: true mean is not equal to 0

95 percent confidence interval:

1.967723 7.874529

sample estimates:

mean of x

4.921126

Leg.trunk_left._Sagittalflex and RT_RECT.ABDOM.LO..uV

One Sample t-test

data: x

t = 2.7446, df = 29, p-value = 0.01029

alternative hypothesis: true mean is not equal to 0

95 percent confidence interval:

0.3583959 2.4544892

sample estimates:

mean of x

1.406443

Leg.trunk_left._Sagittalflex and RT_RECTUS_FEM..uV

One Sample t-test

data: x

t = -2.6665, df = 29, p-value = 0.01240

alternative hypothesis: true mean is not equal to 0

95 percent confidence interval:

-2.0781506 -0.2740048

sample estimates:

mean of x

-1.176078

Leg.trunk_left._Sagittalflex and RT_POST.DELTOID.uV

One Sample t-test

data: x

t = -3.1888, df = 29, p-value = 0.003414

alternative hypothesis: true mean is not equal to 0

95 percent confidence interval:

-6.988038 -1.526832

sample estimates:

mean of x

-4.257435

Leg.trunk_left._Sagittalflex and RT_LUMBAR_ES.uV

One Sample t-test

data: x

t = -3.5892, df = 29, p-value = 0.001205

alternative hypothesis: true mean is not equal to 0

95 percent confidence interval:

-8.470386 -2.321067

sample estimates:

mean of x

-5.395727

Leg.trunk_left._Sagittalflex and LT_BICEPS_FEM..uV

One Sample t-test

data: x

t = -2.3238, df = 29, p-value = 0.02735

alternative hypothesis: true mean is not equal to 0

95 percent confidence interval:

-3.2811913 -0.2091919

sample estimates:

mean of x

-1.745192

Leg.trunk_right._Sagittalflex and Leg.trunk_left._Lateralflex

One Sample t-test

data: x

t = -0.7588, df = 29, p-value = 0.4541

alternative hypothesis: true mean is not equal to 0

95 percent confidence interval:

-2.438315 1.118602

sample estimates:

mean of x

-0.6598563

Leg.trunk_right._Sagittalflex and Leg.trunk_right._Lateralflex

One Sample t-test

data: x

t = 0.27, df = 29, p-value = 0.789

alternative hypothesis: true mean is not equal to 0

95 percent confidence interval:

-1.768587 2.306614

sample estimates:

mean of x

0.2690137

Leg.trunk_right._Sagittalflex and Left_knee._Flex

One Sample t-test

data: x

t = -1.6466, df = 29, p-value = 0.1104

alternative hypothesis: true mean is not equal to 0

95 percent confidence interval:

-7.4787149 0.8074604

sample estimates:

mean of x

-3.335627

Leg.trunk_right._Sagittalflex and RT_PECT._MAJOR.uV

One Sample t-test

data: x

t = 3.2501, df = 29, p-value = 0.002919

alternative hypothesis: true mean is not equal to 0

95 percent confidence interval:

1.834596 8.062872

sample estimates:

mean of x

4.948734

Leg.trunk_right._Sagittalflex and RT_RECT.ABDOM.LO..uV

One Sample t-test

data: x

t = 2.5102, df = 29, p-value = 0.01790

alternative hypothesis: true mean is not equal to 0

95 percent confidence interval:

0.2211333 2.1662960

sample estimates:

mean of x

1.193715

Leg.trunk_right._Sagittalflex and RT_RECTUS_FEM..uV

One Sample t-test

data: x

t = -2.6633, df = 29, p-value = 0.01250

alternative hypothesis: true mean is not equal to 0

95 percent confidence interval:

-2.4140970 -0.3169015

sample estimates:

mean of x

-1.365499

Leg.trunk_right._Sagittalflex and RT_POST.DELTOID.uV

One Sample t-test

data: x

t = -3.0187, df = 29, p-value = 0.005248

alternative hypothesis: true mean is not equal to 0

95 percent confidence interval:

-7.230887 -1.390039

sample estimates:

mean of x

-4.310463

Leg.trunk_right._Sagittalflex and RT_LUMBAR_ES.uV

One Sample t-test

data: x

t = -3.5171, df = 29, p-value = 0.001457

alternative hypothesis: true mean is not equal to 0

95 percent confidence interval:

-8.701306 -2.302522

sample estimates:

mean of x

-5.501914

Leg.trunk_right._Sagittalflex and LT_BICEPS_FEM..uV

One Sample t-test

data: x

t = -2.0896, df = 29, p-value = 0.04553

alternative hypothesis: true mean is not equal to 0

95 percent confidence interval:

-3.19890286 -0.03433704

sample estimates:

mean of x

-1.61662

Leg.trunk_left._Lateralflex and Leg.trunk_right._Lateralflex

One Sample t-test

data: x

t = 8.7453, df = 29, p-value = 1.26e-09

alternative hypothesis: true mean is not equal to 0

95 percent confidence interval:

27.51595 44.31462

sample estimates:

mean of x

35.91528

Leg.trunk_left._Lateralflex and Left_knee._Flex

One Sample t-test

data: x

t = -0.1833, df = 29, p-value = 0.8559

alternative hypothesis: true mean is not equal to 0

95 percent confidence interval:

-2.775765 2.319211

sample estimates:

mean of x

-0.2282774

Leg.trunk_left._Lateralflex and RT_PECT._MAJOR.uV

One Sample t-test

data: x

t = 0.3063, df = 29, p-value = 0.7616

alternative hypothesis: true mean is not equal to 0

95 percent confidence interval:

-1.414962 1.913353

sample estimates:

mean of x

0.2491953

Leg.trunk_left._Lateralflex and RT_RECT.ABDOM.LO..uV

One Sample t-test

data: x

t = 0.8741, df = 29, p-value = 0.3892

alternative hypothesis: true mean is not equal to 0

95 percent confidence interval:

-2.168198 5.405015

sample estimates:

mean of x

1.618408

Leg.trunk_left._Lateralflex and RT_RECTUS_FEM..uV

One Sample t-test

data: x

t = -0.4392, df = 29, p-value = 0.6638

alternative hypothesis: true mean is not equal to 0

95 percent confidence interval:

-2.632591 1.701835

sample estimates:

mean of x

-0.465378

Leg.trunk_left._Lateralflex and RT_POST.DELTOID.uV

One Sample t-test

data: x

t = -1.746, df = 29, p-value = 0.0914

alternative hypothesis: true mean is not equal to 0

95 percent confidence interval:

-3.5626116 0.2811985

sample estimates:

mean of x

-1.640707

Leg.trunk_left._Lateralflex and RT_LUMBAR_ES.uV

One Sample t-test

data: x

t = -0.3976, df = 29, p-value = 0.6939

alternative hypothesis: true mean is not equal to 0

95 percent confidence interval:

-2.402153 1.620225

sample estimates:

mean of x

-0.3909640

Leg.trunk_left._Lateralflex and LT_BICEPS_FEM..uV

One Sample t-test

data: x

t = 2.7814, df = 29, p-value = 0.009412

alternative hypothesis: true mean is not equal to 0

95 percent confidence interval:

0.6119391 4.0118954

sample estimates:

mean of x

2.311917

Leg.trunk_right._Lateralflex and Left_knee._Flex

One Sample t-test

data: x

t = 0.8163, df = 29, p-value = 0.421

alternative hypothesis: true mean is not equal to 0

95 percent confidence interval:

-1.253218 2.917946

sample estimates:

mean of x

0.8323638

Leg.trunk_right._Lateralflex and RT_PECT._MAJOR.uV

One Sample t-test

data: x

t = 0.5452, df = 29, p-value = 0.5898

alternative hypothesis: true mean is not equal to 0

95 percent confidence interval:

-1.173223 2.026061

sample estimates:

mean of x

0.4264191

Leg.trunk_right._Lateralflex and RT_RECT.ABDOM.LO..uV

One Sample t-test

data: x

t = 0.3145, df = 29, p-value = 0.7554

alternative hypothesis: true mean is not equal to 0

95 percent confidence interval:

-2.420454 3.300256

sample estimates:

mean of x

0.4399011

Leg.trunk_right._Lateralflex and RT_RECTUS_FEM..uV

One Sample t-test

data: x

t = 0.4041, df = 29, p-value = 0.6891

alternative hypothesis: true mean is not equal to 0

95 percent confidence interval:

-1.226451 1.830497

sample estimates:

mean of x

0.3020228

Leg.trunk_right._Lateralflex and RT_POST.DELTOID.uV

One Sample t-test

data: x

t = -1.6061, df = 29, p-value = 0.1191

alternative hypothesis: true mean is not equal to 0

95 percent confidence interval:

-2.7626284 0.3322154

sample estimates:

mean of x

-1.215206

Leg.trunk_right._Lateralflex and RT_LUMBAR_ES.uV

One Sample t-test

data: x

t = -0.7498, df = 29, p-value = 0.4594

alternative hypothesis: true mean is not equal to 0

95 percent confidence interval:

-3.977038 1.843182

sample estimates:

mean of x

-1.066928

Leg.trunk_right._Lateralflex and LT_BICEPS_FEM..uV

One Sample t-test

data: x

t = 3.1174, df = 29, p-value = 0.004094

alternative hypothesis: true mean is not equal to 0

95 percent confidence interval:

0.6016558 2.8970066

sample estimates:

mean of x

1.749331

Left_knee._Flex and RT_PECT._MAJOR.uV

One Sample t-test

data: x

t = -2.2894, df = 29, p-value = 0.02952

alternative hypothesis: true mean is not equal to 0

95 percent confidence interval:

-3.3111859 -0.1865496

sample estimates:

mean of x

-1.748868

Left_knee._Flex and RT_RECT.ABDOM.LO..uV

One Sample t-test

data: x

t = -1.5225, df = 29, p-value = 0.1387

alternative hypothesis: true mean is not equal to 0

95 percent confidence interval:

-2.6949972 0.3948404

sample estimates:

mean of x

-1.150078

Left_knee._Flex and RT_RECTUS_FEM..uV

One Sample t-test

data: x

t = 2.0099, df = 29, p-value = 0.05383

alternative hypothesis: true mean is not equal to 0

95 percent confidence interval:

-0.03583463 4.11292640

sample estimates:

mean of x

2.038546

Left_knee._Flex and RT_POST.DELTOID.uV

One Sample t-test

data: x

t = 2.3549, df = 29, p-value = 0.02551

alternative hypothesis: true mean is not equal to 0

95 percent confidence interval:

0.464420 6.599723

sample estimates:

mean of x

3.532071

Left_knee._Flex and RT_LUMBAR_ES.uV

One Sample t-test

data: x

t = 2.2649, df = 29, p-value = 0.03117

alternative hypothesis: true mean is not equal to 0

95 percent confidence interval:

0.2855875 5.6039136

sample estimates:

mean of x

2.944751

Left_knee._Flex and LT_BICEPS_FEM..uV

One Sample t-test

data: x

t = -0.0605, df = 29, p-value = 0.9522

alternative hypothesis: true mean is not equal to 0

95 percent confidence interval:

-1.996829 1.882145

sample estimates:

mean of x

-0.05734193

RT_PECT._MAJOR.uV and RT_RECT.ABDOM.LO..uV

One Sample t-test

data: x

t = 0.7561, df = 29, p-value = 0.4557

alternative hypothesis: true mean is not equal to 0

95 percent confidence interval:

-0.7381949 1.6041634

sample estimates:

mean of x

0.4329842

RT_PECT._MAJOR.uV and RT_RECTUS_FEM..uV

One Sample t-test

data: x

t = -2.1779, df = 29, p-value = 0.03769

alternative hypothesis: true mean is not equal to 0

95 percent confidence interval:

-2.5954785 -0.0815667

sample estimates:

mean of x

-1.338523

RT_PECT._MAJOR.uV and RT_POST.DELTOID.uV

One Sample t-test

data: x

t = -1.6621, df = 29, p-value = 0.1073

alternative hypothesis: true mean is not equal to 0

95 percent confidence interval:

-18.733826 1.935845

sample estimates:

mean of x

-8.39899

RT_PECT._MAJOR.uV and RT_LUMBAR_ES.uV

One Sample t-test

data: x

t = -3.1211, df = 29, p-value = 0.004056

alternative hypothesis: true mean is not equal to 0

95 percent confidence interval:

-5.809431 -1.209815

sample estimates:

mean of x

-3.509623

RT_PECT._MAJOR.uV and LT_BICEPS_FEM..uV

One Sample t-test

data: x

t = 1.0024, df = 29, p-value = 0.3245

alternative hypothesis: true mean is not equal to 0

95 percent confidence interval:

-1.226832 3.585284

sample estimates:

mean of x

1.179226

RT_RECT.ABDOM.LO..uV and RT_POST.DELTOID.uV

One Sample t-test

data: x

t = -1.1849, df = 29, p-value = 0.2457

alternative hypothesis: true mean is not equal to 0

95 percent confidence interval:

-2.3514072 0.6263246

sample estimates:

mean of x

-0.8625413

RT_RECT.ABDOM.LO..uV and RT_LUMBAR_ES.uV

One Sample t-test

data: x

t = -1.944, df = 29, p-value = 0.06165

alternative hypothesis: true mean is not equal to 0

95 percent confidence interval:

-1.67571997 0.04250376

sample estimates:

mean of x

-0.8166081

RT_RECT.ABDOM.LO..uV and LT_BICEPS_FEM..uV

One Sample t-test

data: x

t = -1.5058, df = 29, p-value = 0.1429

alternative hypothesis: true mean is not equal to 0

95 percent confidence interval:

-4.0720646 0.6185974

sample estimates:

mean of x

-1.726734

RT_RECTUS_FEM..uV and RT_POST.DELTOID.uV

One Sample t-test

data: x

t = 4.9216, df = 29, p-value = 3.154e-05

alternative hypothesis: true mean is not equal to 0

95 percent confidence interval:

2.868165 6.946896

sample estimates:

mean of x

4.907531

RT_RECTUS_FEM..uV and RT_LUMBAR_ES.uV

One Sample t-test

data: x

t = 2.9152, df = 29, p-value = 0.006787

alternative hypothesis: true mean is not equal to 0

95 percent confidence interval:

0.6684037 3.8111144

sample estimates:

mean of x

2.239759

RT_RECTUS_FEM..uV and LT_BICEPS_FEM..uV

One Sample t-test

data: x

t = -1.0706, df = 29, p-value = 0.2932

alternative hypothesis: true mean is not equal to 0

95 percent confidence interval:

-1.7631054 0.5515107

sample estimates:

mean of x

-0.6057973

RT_POST.DELTOID.uV and RT_LUMBAR_ES.uV

One Sample t-test

data: x

t = 4.3295, df = 29, p-value = 0.0001625

alternative hypothesis: true mean is not equal to 0

95 percent confidence interval:

2.500059 6.977013

sample estimates:

mean of x

4.738536

RT_POST.DELTOID.uV and LT_BICEPS_FEM..uV

One Sample t-test

data: x

t = -1.0634, df = 29, p-value = 0.2964

alternative hypothesis: true mean is not equal to 0

95 percent confidence interval:

-2.2699537 0.7169683

sample estimates:

mean of x

-0.7764927

RT_LUMBAR_ES.uV and LT_BICEPS_FEM..uV

One Sample t-test

data: x

t = 3.0165, df = 29, p-value = 0.005277

alternative hypothesis: true mean is not equal to 0

95 percent confidence interval:

0.4026207 2.0982768

sample estimates:

mean of x

1.250449
